# Detection and Characterization of *Wolbachia* Infections in Natural Populations of Aphids: Is the Hidden Diversity Fully Unraveled?

**DOI:** 10.1371/journal.pone.0028695

**Published:** 2011-12-13

**Authors:** Antonis A. Augustinos, Diego Santos-Garcia, Eva Dionyssopoulou, Marta Moreira, Aristeidis Papapanagiotou, Marios Scarvelakis, Vangelis Doudoumis, Silvia Ramos, Antonio F. Aguiar, Paulo A. V. Borges, Manhaz Khadem, Amparo Latorre, George Tsiamis, Kostas Bourtzis

**Affiliations:** 1 Department of Environmental and Natural Resources Management, University of Ioannina, Agrinio, Greece; 2 Institut Cavanilles de Biodiversitat i Biologia Evolutiva, Universitat de València, Valencia, Spain; 3 ISOPlexis Gene Bank, Universidade da Madeira, Funchal, Portugal; 4 Department of Greenhouse Crops and Floriculture, Technological Educational Institute of Messolonghi, Messolonghi, Greece; 5 Laboratório de Qualidade Agrícola, Núcleo de Fitopatologia,, Madeira, Portugal; 6 Departamento de Ciências Agrárias CITA-A (Azorean Biodiversity Group), Universidade dos Açores, Angra do Heroísmo, Terceira – Azores; 7 Área de Genómica y Salud, Centro Superior de Investigación en Salud Pública (CSISP), Valencia, Spain; 8 Biomedical Sciences Research Center Al. Fleming, Vari, Greece; 9 Department of Environmental and Natural Resources Management, University of Western Greece, Agrinio, Greece; University of Poitiers, France

## Abstract

Aphids are a serious threat to agriculture, despite being a rather small group of insects. The about 4,000 species worldwide engage in highly interesting and complex relationships with their microbial fauna. One of the key symbionts in arthropods is *Wolbachia*, an α-Proteobacterium implicated in many important biological processes and believed to be a potential tool for biological control. Aphids were thought not to harbour *Wolbachia*; however, current data suggest that its presence in aphids has been missed, probably due to the low titre of the infection and/or to the high divergence of the *Wolbachia* strains of aphids. The goal of the present study is to map the *Wolbachia* infection status of natural aphids populations, along with the characterization of the detected *Wolbachia* strains. Out of 425 samples from Spain, Portugal, Greece, Israel and Iran, 37 were found to be infected. Our results, based mainly on 16S rRNA gene sequencing, indicate the presence of two new *Wolbachia* supergroups prevailing in aphids, along with some strains belonging either to supergroup B or to supergroup A.

## Introduction

*Wolbachia* is a diverse group of obligatory intracellular and maternally transmitted α-Proteobacteria [1]–[3]. Several studies suggest that these bacteria are present in at least 65% of arthropod species as well as in filarial nematodes and in some plant parasitic nematodes [4]–[8]. *Wolbachia* strains infecting arthropod and nematode hosts are represented by a single species, *Wolbachia pipientis* [9]; however, there is extensive diversity which has resulted in the assignment of the bacterial strains into at least eleven *Wolbachia* supergroups, named A to F and H to L (supergroup G is considered a recombinant between A and B) [4], [10]–[19].*Wolbachia* diversity was initially characterized using the genes *wsp*, 16S rRNA, *ftsZ*, *gltA* and *groEL* as molecular markers, while strain genotyping is based on multi locus sequence typing systems (MLST), as well as on the amino acid sequences of the four hypervariable regions (HVRs) of the WSP protein [20], [21].

*Wolbachia* have been reported in the somatic tissues of arthropod hosts; however, they mainly reside in the reproductive tissues and organs [2]. This tissue localization pattern has been associated with the induction of different reproductive alterations such as feminization, parthenogenesis, male killing and cytoplasmic incompatibility [2], [22], which aid the spread of *Wolbachia* infections in host populations [23]. The widespread distribution of *Wolbachia* and their ability to manipulate the reproductive properties of arthropod hosts has attracted interest in its role in host biology, ecology and evolution, as well as in the development of novel, symbiont-based and environment friendly *Wolbachia*-based methods for pest and disease management [2], [3], [24]–[26]. It has been suggested that *Wolbachia*-induced cytoplasmic incompatibility can be used either for the control of agricultural pests and disease vectors through the Incompatible Insect Technique (IIT), or by spreading a desirable genotype through populations, such as the inability of a vector species to transmit a pathogen [27]–[33]. The introduction of life-shortening *Wolbachia* strains could modify the population age structure of insect vector species, thus reducing pathogen transmission [34], [35]. Furthermore, recent studies provide evidence that the presence of *Wolbachia* in some insect species may provide anti-viral protection as well as inhibit the infection with and transmission of certain pathogens such as Dengue, Chikungunya and *Plasmodium* [35]–[40].

Aphids are a rather small group of insects but their threat to agricultural ecosystems is enormous. Currently, there are about 4,000 recognized species worldwide [41]. Aphids do great damage to their host plants in several ways [42]. They feed on plant sap and inject saliva (which can be phytotoxic) during feeding. Their honeydew is used by saprophytic ascomycetes that grow on plants. More importantly, aphids have been shown to be vectors of numerous plant viruses. Due to their feeding behavior, they are by far the most important virus vectors, transmitting ∼30% of all plant virus species [43].

Aphids exhibit many interesting biological traits. They have a complicated life cycle, being able to reproduce both sexually and asexually. They are specialized in probing and using phloem sap as sole food source, which leads to a tight association with their host plants. They are also important for the feeding of other insects; they modify phloem sap, which has a high ratio of non-essential to essential amino acids and elevated sugar content, and produce substances more suitable for other species [44].

Aphids have established sophisticated symbiotic relationships and many of their unique properties can be attributed to their symbiotic bacteria [45]. They have established an obligate mutualistic symbiosis with *Buchnera aphidicola*, whichprovides them with essential amino acids lacking from their phloem diet [46]–[49]. Occasionally, aphids harbour secondary or facultative symbionts that coexist with *Buchnera*, and can have positive effects on the aphid host [45]. It has been reported that ‘*CandidatusHamiltonella defensa*’ and ‘*CandidatusRegiella insecticola*’can protect aphids against parasitoids [50], [51], whereas *CandidatusSerratia symbiotica*is implicated in heat tolerance [52]. Finally, studies showing lateral gene transfer from secondary symbionts to their aphid host and the fact that these genes are expressed in some cases [53], [54], along with a reported case of metabolic complementation between *B. aphidicola* and “*Ca S. symbiotica*” in the aphid *Cinara cedri* [55], [56] illustrate the very complex relationship between aphids and their symbionts. All the above suggest that aphids, together with their host plants and their microbial fauna, not only constitute an interesting biological model worth investigating, but that it is furthermore crucial to study and understand these relationships in order to devise appropriate control methods for these species and the plant diseases they transmit.

A small number of studies has investigated the presence of *Wolbachia* in aphids [6], [57]–[60]. Most of them failed to detect *Wolbachia* [57]–[59]. The first report of aphids (*Toxoptera citricidus*and *Aphis craccivora*) harboring *Wolbachia* was based on Long-PCR and the sequencing of the *wsp* gene [6]. Stronger evidence for the presence of *Wolbachia* in aphid species was based on 16S rDNA sequencing, electron microscopy and *in situ* localization of this endosymbiont in *C. cedri* [60]. It was recently reported that Chinese natural populations of the wheat aphid, *Sitobion miscanthi*, harbour single and/or double *Wolbachia* infections belonging to the A and B supergroup [61].

We undertook extensive screening and report here on the presence of *Wolbachia* infections in natural populations of aphid species. The characterization of these *Wolbachia* strains is based on the use of gene markers 16S rRNA, *ftsZ*, *gltA*, *groEL*, *wsp* and MLST. Our study suggests that neither the detection nor the unraveling of *Wolbachia* diversity in the aphid fauna is an easy task; they demand the development of novel tools.

## Results

### Screening for *Wolbachia* infections in natural populations of aphids

A total of 425 natural samples of aphids were screened for the presence of *Wolbachia* with a *16S rRNA*-based PCR approach using the wspecF/wspecR set of primers (Figure S1). The samples were collected in five countries (Greece, Spain, Portugal, Israel and Iran) and on a variety of host plants (at least 165 different species). Collections were in some cases diachronic. This collection represents 144 different aphid species within 69 genera ofnine subfamilies of the family Aphididae (Aphidinae, Chaitophorinae, Pterocommatinae, Myzocallidinae, Drepanosiphinae, Thelaxinae, Lachninae, Mindarinae and Eriosomatinae) (Table S1). The majority of samples screened belong to the subfamily Aphidinae (tribes Aphidini and Macrosiphini)followed by the subfamily Lachninae (mainly from the Eulachnini tribe), considered by recent studies as the most basal lineage among the aphid subfamilies [62],. and from all three tribes of Eriosomatinae subfamily (Pemphigini, Eriosomatini and Fordini).

The results of the screen, which are presented in Table 1, show that the prevalence of *Wolbachia* infections varied significantly between different aphid populations and can be summarized as follows: (a) *Wolbachia* infection was detected in only 37 out of 425 aphid populations tested; (b) *Wolbachia* was detected in aphid species of the subfamilies Lachninae, Aphidinae, Chaitophorinae Eriosomatinae, and Drepanosiphinae, while no infection was found in the rest of subfamilies; (c) at least eight species of the Lachninae subfamily were found infected including seven *Cinara* species (*C. fresai*, *C. maritimae*, *C. juniperi*, *C. pinea*,*C. tujafilina*, *C. cedri* and *Cinara* sp. from the Eulachnini tribe, *Tuberolachnussalignus* and *Maculolachnus submacula* from the Lachninitribe; (d) at least eleven species of the Aphidinae subfamily were found to be infected; nine of them belong to the Aphidini tribe, including three *Aphis* species (*A. fabae*, *A. nerii* and *A. hederae* ), three samples assigned as *Aphis* sp., and two *Toxoptera* species (*T. auranti* and *T. citricidus*). The remaining belong to the Macrosiphini tribe, two samples assigned as *Cavariella* sp., *Macrosiphum euphorbiae*, *Metopolophium dirhodum* and *Aulacorthum solani*; (e) a single infected species belongs to the Chaitophorinae subfamily (*Sipha maydis*); (f) a single sample of the Eriosomatinae subfamily, *Baizongia pistaciae* (tribe Fordini), was found to harbour *Wolbachia*; and (g) a single sample of the Drepanosiphinae subfamily, *Neophyllaphis podocarpi*, was found to harbour *Wolbachia*.

**Table 1:** Aphid populations positive for *Wolbachia* and PCR amplification results for 16S rDNA, MLST, *wsp*, *gltA* and *groEL* genes.

Sample	Aphid species	Host	16S rRNA	MLST genes						Other genes	
				*gatB*	*coxA*	*ftsZ*	*hcpA*	*fbpA*	*wsp*	*gltA*	*groEL*
**Metazoa; Arthropoda; Insecta; Hemiptera; Sternorrhyncha; Aphididae; Lachninae; Lachnini**											
CS_Valencia9-SP	*Tuberolachnus salignus*	*Salix sp.*	+^1^^,^ 3	−	−	−	−	−	−	−	−
CS_Valencia(Tsa) –SP	*T. salignus*	*Salix sp.*	+^1^^,^ 3	−	+	−	−	−	−	−	−
09Md 24	*T. salignus*	*Salix canariensis*	+^1^^,^ 3 *	−	−	−	−	−	−	−	−
BS_Valencia(Msu) –SP	*Maculolachnus submacula*	*Rosa sp.*	+^1^^,^ 3 *	+	−	−	−	−	−	−	−
**Metazoa; Arthropoda; Insecta; Hemiptera; Sternorrhyncha; Aphididae; Lachninae; Eulachnini**											
09Madeira23-PO	*Cinara fresai*	*Cupressus macrocarpa*	+^1^^,^ 3 *	−	−	−	−	−	−	−	−
CS_Valencia2-SP	*Cinara maritimae*	*Pinus pinaster*	+^1^^,^ 3	−	−	−	−	−	−	−	−
CS_Valencia3-SP	*Cinara juniperi*	*Juniperus communis*	+^1^^,^ 3	−	−	−	−	−	−	−	−
CS_Valencia4-SP	*Cinara pinea*	*Pinus sylvestris*	+^1^^,^ 3	−	−	−	−	−	−	−	−
CS_Valencia7-SP	*Cinara tujafilina*	*Platycladus orientalis*	+^1^^,^ 3 *	−	−	−	−	−	−	−	−
09Madeira48-PO	*Cinara pinea*	*Pinus sp.*	+^1^^,^ 3	−	−	−	−	−	−	−	−
AS_Valencia(CCeV-SP)	*Cinara cedri*	*Cedrus sp.*	+^1^^,^ 3	−	−	−	+	−	+	−	+
BS_Galicia(CCeG) –SP	*Cinara cedri*	*Cedrus sp.*	+^1^^,^ 3	−	−	−	−	−	+	−	−
BS_Salamanca(CCeS) –SP	*Cinara cedri*	*Cedrus sp.*	+^1^^,^ 3	+	+	+	−	−	+	+	+
BS_Tarancon(CCeT) –SP	*Cinara cedri*	*Cedrus sp.*	+^1^^,^ 3	+	+	−	−	−	−	+	+
BS_Zaragoza(CCeZ) –SP	*Cinara cedri*	*Cedrus sp.*	+^1^^,^ 3	+	+	−	+	+	+	+	+
CS_Valencia (CCeV)-SP	*Cinara cedri*	*Cedrus sp.*	+^1^^,^ 3	−	+	−	−	−	+	−	−
10Iran12	*Cinara sp.*	*Cupressus sp.*	+^1^^,^ 3	+	+	−	−	−	−	−	−
BS_Israel(CCeI w^−^)	*Cinara cedri*	*Cedrus sp.*	+^1^^,^ 3	−	+	−	−	−	−	−	−
BS_Israel(CCeI w^+^)	*Cinara cedri*	*Cedrus sp.*	+^1^^,^ 3	−	−	−	−	−	−	−	−
10Madeira181-PO	*Cinara pinea*	*Pinus sp.*	+^2^^,^ 3 *	−	−	−	−	−	−	−	−
10Iran3	*Cinara sp.*	*Pinus sp.*	+^1^^,^ 3 *	−	−	−	−	−	−	−	−
**Metazoa; Arthropoda; Insecta; Hemiptera; Sternorrhyncha; Aphididae; Aphidinae; Macrosiphini**											
CS_Valencia1-SP	*Cavariella sp.*	*Salix sp.*	+^1^^,^ 3	−	−	−	−	−	−	−	−
BS_Valencia-SP	*Cavariella sp.*	*Salix sp.*	+^1^^,^ 3	+	−	−	−	−	−	−	+
GRA40	*Metopolophium dirhodum*	*T. aestivum*	^−^	+	+	−	−	−	+	−	−
11Md 199	*Aulacorthum solani*	*Euphorbia piscatoria*	+^1^^,^ 3 *	−	−	−	−	−	−	−	−
11Md 203	*Macrosiphum euphorbiae*	*Solandra grandiflora*	+^1^^,^ 3 *	−	−	−	−	−	−	−	−
**Metazoa; Arthropoda; Insecta; Hemiptera; Sternorrhyncha; Aphididae; Aphidinae; Aphidini**											
GRA4	*Aphis fabae*	*Phaseolus vulgaris*	^−^	−	−	−	−	+	+	−	−
CS_Valencia6-Sp	*Aphis nerii*	*Nerium oleander*	+^1^^,^ 3 *	−	−	−	−	−	−	−	−
CS_Valencia8-Sp	*Aphis sp.*	*Genista sp.*	+^1^^,^ 3 *	−	−	−	−	−	−	−	−
GRA17	*Aphis hederae*	*Hedera helix*	+^2^^,^ 3 *	−	−	−	−	−	−	−	−
10Az16	*Aphis sp.*	*Nerium oleander*	+^1^^,^ 3 *	−	−	−	−	−	−	−	−
10Az10	*Aphis sp.*	*Strelitzia sp.*	+^1^^,^ 3 *	−	−	−	−	−	−	−	−
10Madeira187-PO	*Toxoptera citricida*	*Annonaceae*	+^2^^,^ 3 *	+	−	−	−	+	−	−	−
10Az3	*Toxoptera aurantii*	*Agapanthus sp.*	+^1^^,^ 3 *	−	−	−	−	−	−	−	−
**Metazoa; Arthropoda; Insecta; Hemiptera; Sternorrhyncha; Aphididae; Chaitophorinae; Siphini**											
GCC201	*Sipha maydis*	Gramineae	+^2^^,^ 3 *	+	−	−	−	−	−	−	−
**Metazoa; Arthropoda; Insecta; Hemiptera; Sternorrhyncha; Aphididae; Eriosomatinae; Fordini**											
GRA69	*Baizongia pistaciae*	*Pistacia terebinthus*	+^2^^,^ 3 *	+	−	+	−	+	−	−	−
**Metazoa; Arthropoda; Insecta; Hemiptera; Sternorrhyncha; Aphididae; Drepanosiphinae**											
10AzG3	*Neophyllaphis podocarpi*	*Podocarpus macrophylus*	+^1^ *	−	−	−	−	−	−	−	−

+: amplification,

1 Nested PCR: first set 16S-169F/1513R, second set 16S-169F/WspecR,

2 169F/16S_woR1,

3 wspecF/wspecR.

*Cloned on pGEM and sequenced with Sp6/T7 universal primers, −: failure to detect amplification product.

It should be noted that at least four different individuals of *Cinara pinea* (Madeira), *Metopolophium dirhodum*, *Aphis fabae*, *Aphis hederae*, *Toxoptera citricidus* (Madeira), *Sipha maydis* and *Baizongia pistaciae* populations were tested. All individuals were *Wolbachia* positive. For the rest of the populations, the screening was performed on a pool of four individuals.

Taken together, these results suggest that *Wolbachia* may be more abundant in aphids than previously thought, and that new universal primers coupled with new sequencing technologies will enable a better detection and investigation of the *Wolbachia* diversity.

### Genotyping aphid *Wolbachia* strains

The current genotyping of *Wolbachia* strains is based on MLST approaches [20], [21]. Efforts were made to amplify the MLST genes for the *Wolbachia*-infected aphid samples; however, the majority of PCRs failed. Only for a few of the samples, some of the genes were successfully amplified (Table 1). Due to these difficulties, attempts were undertaken to characterize the bacterial strains present in each of the thirty-seven *Wolbachia*-infected aphid populations using a near-full length sequence of the 16S rRNA gene. Additional markers were also used, such as *groEL,gltA, wsp* and/or other individual MLST gene markers(*gatB, coxA, ftsZ, hcpA and fbpA*), which could be amplified from the *Wolbachia*-infected aphid samples.

The results of these efforts can be summarized as follows: (a) a near-full length sequence of the 16S rRNA gene was amplified and analyzed for 35 out of the 37 *Wolbachia*-infected aphid samples, using PCR-sequencing approaches and the primers as presented in Table 1 and Figure S1. For two samples, GRA4 and GRA40, the amplification of 16S rRNA gene was not possible, and the characterization was based on other genes (see Table 1); (b) genes *gatB*, *coxA*, *ftsZ*, *hcpA*, *fbpA*, *wsp*, *gltA* and *groEL* were amplified only from ten, eight, two, two, four, seven, three and five *Wolbachia*-infected aphid samples, respectively (Table 1); (c) the sequence analysis of *gatB*, *coxA*, *ftsZ*, *hcpA*, *fbpA* and *wsp* revealed the presence of eight, three, two, two, four and two alleles respectively (Tables 2 and 3); (d) the sequence analysis also indicated the presence of novel alleles: seven for *gatB*, one for *ftsZ*, one for *hcpA* and three for *fbpA* (Tables 2 and 3); (e) *gltA* and *groEL* gene fragments were amplified only in three and five *Wolbachia*-infected aphid samples, respectively (Table 1).

**Table 2:** *Wolbachia* MLST allele profiles for positive aphid populations.

Sample	Aphid species	Host	Wolbachia MLST				
			*gatB*	*coxA*	*ftsZ*	*hcpA*	*fbpA*
AS_Valencia(CCeV-SP)	*Cinara cedri*	*Cedrus sp.*	−	−	−	29	−
BS_Salamanca(CCeS) –SP	*Cinara cedri*	*Cedrus sp.*	**160**	87	35	−	−
BS_Tarancon(CCeT) –SP	*Cinara cedri*	*Cedrus sp.*	**161**	87	−	−	−
BS_Zaragoza(CCeZ) –SP	*Cinara cedri*	*Cedrus sp.*	**162**	87	−	**172**	**223**
CS_Valencia (CCeV)-SP	*Cinara cedri*	*Cedrus sp.*	−	87	−	−	−
10Iran12	*Cinara sp.*	*Cupressus sp.*	**163**	87	−	−	−
BS_Israel(CCeI w^−^)	*Cinara cedri*	*Cedrus sp.*	−	84	−	−	−
BS_Valencia-SP	*Cavariella sp.*	*Salix sp.*	161				
BS_Valencia(Tsa) –SP	*Tuberolachnus salignus*	*Salix sp.*	−	1	−	−	−
BS_Valencia(Msu) –SP	*Maculolachnus submacula*	*Rosa sp.*	**164**	−	−	−	−
GRA40	*Metopolophium dirhodum*	*T. aestivum*	8	84	−	−	−
GRA4	*Aphis fabae*	*Phaseolus vulgaris*	−	−	−	−	160
10Madeira187-PO	*Toxoptera citricidus*	*Annonaceae*	**164**	−	−	−	**224**
GCC201	*Sipha maydis*	Gramineae	**165**	−	−	−	−
GRA69	*Baizongia pistaciae*	*Pistacia terebinthus*	**166**	−	**131**	−	**225**

**Table 3:** *Wolbachia* WSP HVR profiles for aphid populations.

Sample	Aphid species	Host	wsp	HRV1	HRV2	HRV3	HRV4
Valencia(CCeV-SP)	*Cinara cedri*	*Cedrus sp.*	584	2	17	3	234
Galicia(CCeG) –SP	*Cinara cedri*	*Cedrus sp.*	584	2	17	3	234
Salamanca(CCeS) –SP	*Cinara cedri*	*Cedrus sp.*	584	2	17	3	234
Zaragoza(CCeZ) –SP	*Cinara cedri*	*Cedrus sp.*	584	2	17	3	234
Valencia (CCeV)-SP	*Cinara cedri*	*Cedrus sp.*	584	2	17	3	234
GRA40	*Metopolophium dirhodum*	*Triticum aestivum*	335	1	12	21	144
GRA4	*Aphis fabae*	*Phaseolus vulgaris*	335	1	12	21	144

These results indicate that there are differences in the *Wolbachia* infection status among different aphid species and populations and, more importantly, that the currently available genotyping tools of *Wolbachia* [10], [20], [21], [63], [64]cannot be universally applied for *Wolbachia* of aphids.

### Phylogenetic analysis

**Figure 1:**
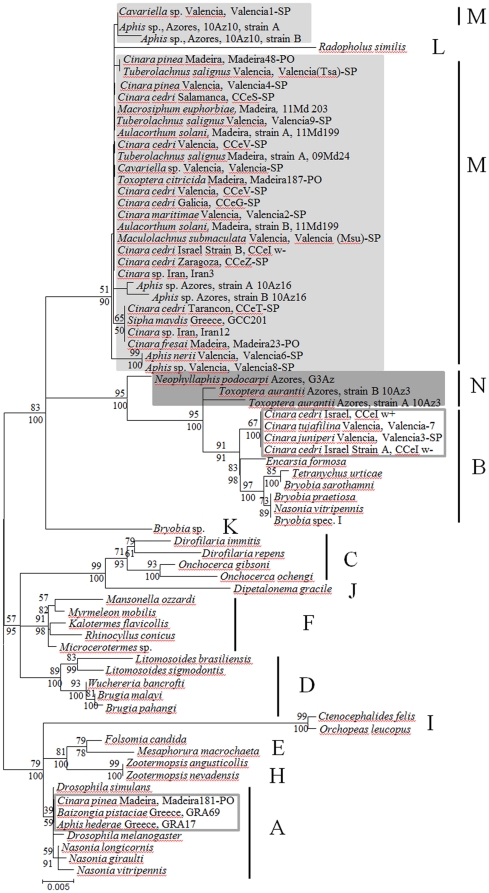
Unrooted phylogenetic tree of *Wolbachia* 16S rRNA gene sequences. Strains are designated with the names of their host species, followed by the collection site and the sample name. Bayesian posterior probabilities (bottom number) and ML bootstrap values based on 1000 replicates are given. *Wolbachia* supergroups are shown to the right of the host species names. New supergroups are shaded while aphid *Wolbachia* strains that belong to supergroup A or B are boxed.

Failure to amplify the majority of the MLST and/or other protein coding genes meant that the phylogenetic analysis had to be based mainly on partial 16S rRNA gene sequences (at least 1100 bp). Our *Wolbachia* sequences appear to cluster in four different groups: few belong either to supergroup A or to supergroup B, while the majority of the sequences form two new clades M and N, distinct from the so far reported supergroups, as depicted in Figure 1. Genetic distances of all the samples of the new groups are more than 2% from the so far reported A to L supergroups (Table S2). The 2% distance is a value necessary for the establishment of a new supergroup [65], [66]. Supergroup M includes 30 new *Wolbachia* sequences and shows the smallest genetic distance to supergroup B (0.021) and the largest distance to supergroup J (0.059) (Table S3). Supergroup N includes 3 new *Wolbachia* sequences and shows the smallest genetic distance to supergroup K (0.022) and the largest to supergroup I (0.054) (Table S3). The - within the group - genetic distances of these new groups are only 0.013 and 0.002 for supergroup M and N, respectively, supporting the classification of the strains in new clades. Given the tree topology, presence of recombination events was also examined between M, N and B supergroups, along with *Wolbachia* strains fromaphids placed into supergroups A and B. No indication of recombination events were detected using the RDP3 package.

A thorough phylogenetic analysis based on protein coding genes could not be completed, due to PCR amplification failure in most of the cases (Table 1); however the phylogenetic analysis that was based on the available aphid *WolbachiagltA*, *gatB*, *fbpA* and *groEL* gene sequences provided several important findings. *gltA*-based data indicate that the three amplified *Wolbachia* sequences belong to Supergroup B, while the corresponding 16S rRNA sequences group with the new supergroup M, which has, as stated above, the smallest genetic distance to supergroup B. Interestingly, all three sequences were amplified from *C. cedri* populations (Figure S2). *gatB* sequence analysis indicates that almost all amplified sequences group together in a new phylogenetic lineage close to that of supergroup B, except one that groups with supergroup A (Figure S3). A similar picture can be seen with the *fbpA*-based data with three *Wolbachia* sequences forming a new phylogenetic cluster and one grouping with supergroup A sequences (Figure S4). For the *groEL*-based data, one sequence makes a new phylogenetic cluster with supergroup L while the other four group with supergroup B sequences (Figure S5).

Taken together, these results suggest that the aphid fauna may contain an unprecedented range of highly diverse *Wolbachia* strains, which requires the development of new tools for their detection. In addition, these data clearly indicate the need for the development of new (MLST) tools for the genotyping of *Wolbachia* strains belonging to new and/or less characterized supergroups.

## Discussion

### Extending our knowledge on *Wolbachia* infection of aphids

The presence of *Wolbachia* was investigated for 425 samples belonging to 153 different species and 70 genera, using a *Wolbachia* specific *16S rRNA*-based PCR assay. The screen included aphid subfamilies with no previous reports of *Wolbachia* infection and included aphid species from different geographic locations and a variety of hosts (at least 165 different species) [6], [57]–[60]. Despite difficulties with PCR amplification (see below), *Wolbachia* infections were detected, adding important information to previous studies on aphids which had detected *Wolbachia* in only four species: three of these species belong to the Aphidinae subfamily and one to the Lachninae. The present analysis showed that the prevalence of *Wolbachia* infections varied significantly between different aphid populations (Table S1). *Wolbachia* were detectedin eighteen new aphid species, belonging to the subfamilies Chaitophorinae, Eriosomatinae and Drepanosiphidae, while they were not found in 146 species tested belonging to the seven aphid subfamilies: Aphidinae, Chaitophorinae, Pterocommatinae, Drepanosiphinae, Lachninae, Mindarinae and Eriosomatinae.

A direct comparison with previous screening efforts is difficult since: (a) aphid hosts, sample origin and even screening approaches differed and (b) not many aphid species were common in these studies. Our study confirmed previous results regarding the absence of *Wolbachia* in members of the subfamily Aphidinae: (i) *Acyrthosiphon pisum* [58], [59]; (ii) different species of *Uroleucon* genus [59]; (iii) *A. craccivora*, *Myzuspersicae*, *Rhopalosiphumpadi*, *Rhopalosiphummaidis* and *Schizaphis graminum* [59] and (iv) *Aphis jacobaeae*, *Capitophorus carduinis* and *Sitobium fragariae* [57]. It should be noted that *Wolbachia* was not detected in any species tested of the genera*Uroleucon*, *Capitophorus*, *Myzus* and *Sitobion* although *Wolbachia* infection was reported in a previous study [61].

Our study also confirmed previous results regarding the absence of *Wolbachia* in *A. craccivora* [6]and the presence [60] in all but one *C. cedri* samples tested (originating from different geographic locations: Spain, Portugal, Iran and Israel). *Wolbachia* were also detected in five more *Cinara* species (*C. pinea*, *C. fresai*, *C. juniperi*, *C. tujafilina* and *C. maritimae*), suggesting that the genus *Cinara* has a well-established symbiotic association with *Wolbachia*. However, it is difficult to speculate about a possible role of *Wolbachia* in this genus because in 20 out of the 37 samples screened, *Wolbachia* was not detected. In any case, most members of the Lachninae subfamily harbor *S. symbiotica* as a second symbiont [67], [68] and, thus the possibility that these species are more prone to accept other infections cannot be ruled out. Finally, the possibility of a co-evolution with the host can be discarded. First, samples from the same species and the same or different location are found in different supergroups (i.e. *C. cedri* from Israel and Valencia, Spain are found in M and B supergroups; samples from *C. pinea* are found in M and A supergroup). Second, due to the fact that several of the *Cinara* species were previously studied in a work analyzing the presence of *Serratia* in the subfamily Lachninae (94), we can compare the phylogenetic tree obtained in the present work, with those of *Buchnera* and *Serratia* previously obtained. The topology obtained regarding the samples from *Cinara* sp is non-congruent either with *Buchnera* or with *Serratia*. A very interesting result is the identification of multiple infections in *C. cedri* samples. PCR-sequencing analysis of 16S rRNA clones from Israeli populations of *C. cedri* indicates the presence of two *Wolbachia* strains: one from supergroup B and a second from the new supergroup M (see below; Figure 1). The fact that DNA was extracted from a mix of four aphids leaves the possibility open that these two strains derive from different individuals.

There are two limitations in our study, regarding the detection of superinfections: the first is the low body mass of many aphid species, which did not allow isolation of high quality and quantity single-aphid DNA for multiple PCRs. The second is the small number of individuals analyzed per population, since we focused on the screening of as many populations as possible, which, in association with the differential abundance of strains and the non-optimized PCR protocols can lead to under-estimation of multiple infections. It should be noted that *Wolbachia* superinfections have repeatedly been reported in different insect taxa, including Chinese populations of the wheat aphid*Sitobion miscanthi* [61], [69]–[75].

### Extending our knowledge on *Wolbachia* diversity - Two new supergroups

The 16S rRNA gene sequence analysis strongly supports the existence of two new *Wolbachia* supergroups in aphids and raises questions about the robustness of supergroups E, F and H (Figure 1). Thirty-three of the aphid *Wolbachia*-specific 16S rRNA gene sequences cluster in two new clades, which are at least 2% genetically distant from all previously described supergroups and from each other (Table S3). However, the analysis also shows that the distance of supergroup A 16S rRNA gene sequences is less than 2% from the sequences present in supergroups E, F and H, suggesting that the overall classification of *Wolbachia* strains in supergroups (A to N) should be re-evaluated (see Table S3, figures in bold).

Our analysis indicates that the within-supergroup diversity of M and N is 1.3% and 0.2%, respectively (Table S3). Similar phylogenetic analysis with the rest of the genes that are currently being used for the designation of supergroups, could not be completed due to failure of most PCR amplifications. However, the analysis performed with the limited available protein encoding gene sequences (*groEL* and some MLST genes) also support the presence of new supergroups (Figures S3, S4 and S5).

Earlier efforts to characterize *Wolbachia* infections were based on the 16S rDNA and *ftsZ* genes, and later *groEL* and *gltA* were included [10], [64], [76]. In 2006, MLST-based systems were proposed for systematic genotyping and strain classification of *Wolbachia* infections [20], [21]. However, the bacterial strains present in 37 *Wolbachia*-infected aphid populations, representing 25 aphid species, could not be genotyped using MLST analysis due to failure of PCR amplification despite great effort (Table 1). We managed to obtain sequences from ten samples for *gatB*, eight for *coxA*, two for *ftsZ*, two for *hcpA*, four for*fbpA*, seven for *wsp*, three for *gltA* and five for *groEL* (Table 1). Although the sequence analysis in the MLST and wsp databases indicated the presence of new alleles ( Tables 2 and 3), it also clearly shows that the currently available tools cannot be applied universally for the genotyping of the highly diverse aphid *Wolbachia* strains, and a new MLST system may need to be developed.

### The challenge of detection and strain classification of *Wolbachia* infections in aphids

A major crossroad will be the choice of genes for a new MLST system, given that in the present study there were two cases [see Table 1: *Aphis fabae* (GRA4) and *Metopolophium dirhodum* (GRA40)] where *Wolbachia*-specific amplicons were obtained and confirmed by sequencing analysis, also for some MLST genes, but not for the 16S *rRNA* gene, which is considered one of the most conserved genes. Our data are in agreement with recent efforts on the assessment of PCR protocols for the detection of *Wolbachia*,which suggested that the current tools are far from optimal [77].

The development of robust and efficient *Wolbachia* detection and classification protocols is certainly hindered by the presence of low titre infections and multiple infections [78]–[80]. It has been reported that *Wolbachia* density may be affected and/or regulated by co-infection with other *Wolbachia* strains or other vertically transmitted symbionts, as well as by host genotype [81]–[83].

Another important factor is horizontal transfer of *Wolbachia* genes to host genomes, which further complicates both *Wolbachia* detection and strain classification. Horizontal transfer events of *Wolbachia* genome fragments have been reported for several invertebrate species [84]–[88].It is evident that such phenomena can complicate phylogenetic analysis, since nuclear gene copies would evolve in a different way than cytoplasmic copies of *Wolbachia* genes. Also, *Wolbachia* detection is compromised in populations that carry nuclear copies of *Wolbachia* genes but lost the cytoplasmic *Wolbachia* [87]. The draft genome sequence of the pea aphid *Acyrthosiphon pisum* revealed the existence of 12 genes of bacterial origin [89], nine of which were intact and closely related to genes of α-Proteobacteria. There is, however, no evidence for horizontal transfer of *Wolbachia* genes in aphids, and *Wolbachia* was neither detected in the pea aphid in the present nor in previous studies [58], [59]. The PCR detection of some MLST genes, but not of the 16S *rRNA* gene in two aphid samples of the present study (*Aphis fabae* (GRA4) and *Metopolophium dirhodum* (GRA40)) could be explained by the integration of genomic sequences of a former *Wolbachia* symbiont into the host genome although alternative causes can not be excluded.

### Possible role of *Wolbachia* in aphids

Aphids feed on phloem sap, which has an unbalanced nitrogen/carbon content and is deficient in a number of nutrients, mainly amino acids, which insects cannot synthesize and are provided by *Buchnera aphidicola*, their primary endosymbiont. The relationship is mutualistic, since aphids need *B. aphidicola* for normal growth and reproduction, whereas the bacteriumcannot live outside the aphid [46], [47], [90]. In addition to *B. aphidicola*, some aphid populations harbor other heritable bacterial symbionts that are not required for host growth and reproduction, referred to as facultative or secondary symbionts [50], [91]. The most common facultative symbionts found in aphids are ‘*Ca.Regiella insecticola*’, ‘*Ca.Hamiltonella defensa*’ and ‘*Ca.Seratia symbiotica*’ [45], [91]. Several studies, mainly in *A. pisum*, a member of the Aphidinae subfamily, have shown that these symbionts can provide some benefits to the host; however, as mentioned above, no *Wolbachia* has so far been detected in *A. pisum*. The genome sequence of these endosymbionts shows that they have lost the ability to synthesize some amino acids and are thus dependent on *Buchnera* [92]–[94].

*C. cedri*, a member of the subfamily Lachninae that possess the *B. aphidicola* with the smallest genome reported so far, and has established a permanent association with the co-primary endosymbiont *Serratia symbotica*, deserves special attention. Both bacteria are needed for the survival of the whole consortium. When *Wolbachia* was found in *C. cedri*, it was postulated that its presence could increase the prevalence of asexual lineages, (*C. cedri* has a cyclic parthenogenetic life cycle) (see below). In the present study, *Wolbachia*has been found in all analyzed *C. cedri* populations, corroborating their tight association with this species.

Facultative endosymbionts are a common feature of the Lachninae subfamily, to which *C. cedri* belongs [67], [68]. These symbionts are somehow compensating the drastic metabolic losses that have occurred in *B. aphidicola* as it has been recently shown for *C. tujafilina* [95]. The presented data indicate that the members of the Lachninae subfamily tend to be infected with *Wolbachia*. The possibility that *Wolbachia* may have a nutritional function in these cases cannot be discarded, as it has been recently proven in thebedbug, *Cimex lectularius* [96].

*Wolbachia* is well known for its ability to induce reproductive alterations, such as parthenogenesis, feminization, male-killing and, most commonly, cytoplasmic incompatibility, in its hosts [2], [3]. Aphids are known to have complicated life cycles, which include sexually and asexually reproducing species, as well as species with both sexual and asexual phases [97]. Whether *Wolbachia* is somehow involved in these phenomena remains to be investigated. Specifically, it would be interesting to check the life cycle of *Wolbachia*-infected versus non-infected aphids, as its presence could increase the prevalence of asexual lineages, as previously reported for the Hymenopteran group [22].

### Conclusion

We report the largest screening effort so far for *Wolbachia* in aphids. Our results indicate the presence of two new supergroups prevailing in aphids, previously well hidden, probably due to low titer, genetic variability and lack of optimized identification and classification tools. Although *Wolbachia* was unambiguously identified only in a fraction of the samples analyzed, we believe that its presence is underestimated, and the development of more universal *Wolbachia*-screening tools is needed. Clarifying the *Wolbachia* status of aphids can help in the development of novel and environment-friendly methods for the efficient control of aphids, major pests and disease vectors.

## Materials and Methods

### Sample collection and DNA extraction

Aphid taxa examined in this study, information about their taxonomy, collection locations and the host plants they have been isolated from are listed in Table S1. Natural aphid populations were sampled in different years in Greece (2006, 2007, 2009), Iran (2009, 2010), Israel (2005), Portugal (2009, 2010, 2011) and Spain (2003, 2005, 2009) from a variety of host plants. Aphid species were identified based on morphological criteria [98]–[101] and were stored in 100% ethanol at −20°C. Total DNA of the Greek aphid populations was extracted from single aphids (at least three individuals per sample) while for the Spanish, Portuguese, Israeli and Iranian samples, extractions were done from a pool of four adults. DNA extraction was performed as described previously [102]or by using a modified CTAB protocol [103].

### PCR screen

A total of 425 specimens from five subfamilies of the 148 different aphid species were screened for the presence of *Wolbachia* strains. Detection was based on the amplification of a 16S rRNA gene fragment (438 base pairs) with the *Wolbachia* specific primers wspecF and wspecR (Figure S1) [5]. For those samples that appeared negative for *Wolbachia* infection, the quality of DNA was further examined by amplifying part of the mitochondrial 12S rRNA gene (420 base pairs) using primers 12SCFR 5′-GAGAGTGACGGGCGATATGT-3′ and 12SCRR 5′-AAACCAGGATTAGATACCCTATTAT-3′ [104]. PCR amplifications were performed in 20 µl reactions containing 1 µl of DNA, 4 µl 5× reaction buffer (Promega), 1.6 µl MgCl_2_ (25 mM), 0.1 µl deoxynucleotide triphosphate mixture (25 mM each), 0.5 µl of each primer (25 µM), 0.1 µl of *Taq* polymerase (Promega, 1 U/µl) and 12.2 µl water. Amplification was performed in a PTC-200 Thermal Cycler (MJ Research), using the following cycling conditions: 95°C for 5 min, followed by 34 cycles of 30 s at 94°C, 30 s at 54°C, 1 min at 72°C and a final extension of 10 min at 72°C. PCR reactions were electrophoresed on a 1.5% agarose gel. Positive samples were further analysed.

### PCR, cloning and sequencing of 16S rRNA, *groEl*, *gltA*, *wsp* and MLST gene fragments

Amplification of near full size *16S rRNA* sequences proved to be a rather difficult task and required the deployment of a number of approaches (see Figure S1). These involved the use of (a) a new *Wolbachia* specific primer, W169F, designed for the purposes of this study and the universal eubacterial primer 1513R, followed by a nested PCR using the same forward primer (W169F) and wspecR and (b) the newly designed primer W169F and the new *Wolbachia* specific primer 16S_woR1 as reverse primer (Figure S1). For some of the populations, a direct PCR with 16S_169F/16S_woR1 was used. PCR amplifications were performed in 20 µl reactions containing 1 µl of DNA, 4 µl 5× reaction buffer (Promega), 1.6 µl MgCl_2_ (25 mM), 0.1 µl deoxynucleotide triphosphate mixture (25 mM each), 0.5 µl of each primer (25 µM), 0.1 µl of *Taq* polymerase (Promega 1 U/µl) and 12.2 µl water. Amplification was performed in a PTC-200 Thermal Cycler (MJ Research), using the following cycling conditions: 95°C for 5 min, followed by 34 cycles of 30 s at 94°C, 30 s at 51°C for W169F/1513R and 53°C for W169F/16SwolR1, 1 min at 72°C and a final extension of 10 min at 72°C. The annealing temperature for the nested PCR was 53°C.

The *Wolbachia* strains of infected aphid populations were genotyped by MLST, *wsp*, *groEL* and *gtlA* based approaches. Gene fragments of the *groEL*, *gtlA,wsp* and the MLST genes (*gatB*, *coxA*, *hcpA*, *fbpA* and *ftsZ*) were amplified using the respective primers reported previously [17], [20], [64].

### Cloning and sequencing

To determine the sequence of 16S rRNA, *wsp*, *groEL*, *gtlA* and MLST gene fragments, PCR fragments were cloned in cases of poor sequencing quality or multiple chromatographic peaks in direct sequencing of PCR products. PCR products from 18 out of the 37 populations harboring *Wolbachia* were ligated into a T-vector (pGEM-T Easy) and then transformed into DH5α competent cells according to the manufacturer's instructions. Four to six clones were directly subjected to PCR using the primers T7 and SP6. The colony PCR products were purified using the PEG-NaCl method [105] or using NucleoFast® 96 PCR Plates (Macherey-Nagel) according to the manufacturer's instructions. Inserts were fully sequenced with the same primers and with the internal 16S rRNA primer 960R [106]. A dye terminator-labelled cycle sequencing reaction was conducted with the BigDye Terminator v3.1 Cycle Sequencing Kit (PE Applied Biosystems). Reaction products were analysed using an ABI PRISM 310 or an ABI 3730 Genetic Analyzer (PE Applied Biosystems).All *Wolbachia* gene sequences generated in this study were assembled and manually edited with SeqManII by DNAStar. For each sample, a majority-rule consensus sequence was created.

### Nucleotide sequence accession numbers

All 16S rRNA, *wsp*, *groEL*, *gtlA* and MLST gene sequences generated in this study have been deposited in the GenBank database under accession numbers JN384025–JN384106.

### Phylogenetic analysis

All *Wolbachia* 16S rRNA, *gatB*, *fbpA*, *hcpA*, *ftsZ*, *coxA*, *groEL* and *gltA* gene sequences generated in this study were aligned using MUSCLE [107] and ClustalW [108]. Sequences obtained from GenBank representing all currently known supergroups of *Wolbachia* were included in the analysis (Table S2). Phylogenetic analyses were performed using maximum-likelihood (ML) and Bayesian methods. PAUP version 4.0b10 was used to select the optimal evolution model by critically evaluating the selected parameters using the Akaike Information Criterion [109]. For the 16S rRNA and *gltA* gene sequence data the submodel GTR+I+G was selected. For the *groEL*, *gatB* and *fbpA* sequence data, the submodel GTR+G was selected. ML analysis was performed in PAUP using a heuristic search with a random addition of sequences with ten replicates and TBR swapping. The robustness was assessed with 1,000 bootstrap replicates. Bayesian analyses were performed as implemented in MrBayes 3.1 [110]. Analyses were initiated from random starting trees. Four separate runs, each composed of four chains were run for 6,000,000 generations. The cold chain was sampled every 100 generations, and the first 20,000 generations were discarded. Posterior probabilities were computed for the remaining trees.

Recombination events were examined with the default options of the RDP3 software package (Heath et al. 2006).To test for recombination events, we used the RDP3 software package, with all available softwares implemented in it [111]. We used the default options for all analyses.

## Supporting Information

Figure S1 **Position of the primers used in this study, relative to the 16S rRNA gene from wMel.**
(TIF)

Figure S2 **Bayesian inference phylogeny based on** ***gltA*** **data.** The three new *Wolbachia* strains are indicated with bold letters, and the other strains represent supergroups A, B, C, D, F, H, I, and K. Strains are designated with the names of their host species, followed by the collection site and the sample name. Bayesian posterior probabilities (top numbers) and ML bootstrap values based on 100 replicates (bottom numbers) are given.
(TIF)

Figure S3 **Bayesian inference phylogeny based on** ***gatB*** **data.** The 10 new *Wolbachia* strains are indicated with bold letters, and the other strains represent supergroups A, B, D, F, and H. Strains are designated with the names of their host species, followed by the collection site and the sample name. Bayesian posterior probabilities (top numbers) and ML bootstrap values based on 100 replicates (bottom numbers) are given.
(TIF)

Figure S4 **Bayesian inference phylogeny based on** ***fbpA*** **data.** The four new *Wolbachia* strains are indicated with bold letters, and the other strains represent supergroups A, B, D, and F. Strains are designated with the names of their host species, followed by the collection site and the sample name. Bayesian posterior probabilities (top numbers) and ML bootstrap values based on 100 replicates (bottom numbers) are given.
(TIF)

Figure S5 **Bayesian inference phylogeny based on** ***groEL*** **data.** The five new *Wolbachia* strains are indicated with bold letters and the other strains represent supergroups A, B, C, D, F, H, I, K, and L. Strains are designated with the names of their host species, followed by the collection site and the sample name. Bayesian posterior probabilities (top numbers) and ML bootstrap values based on 100 replicates (bottom numbers) are given.
(TIF)

Table S1 ***Wolbachia*** **detection of all aphid populations examined in this study, based on 16S rDNA gene sequencing.**
(DOC)

Table S2 **Taxonomic details of** ***Wolbachia*** **hosts and accession numbers of analyzed sequences.**
(DOC)

Table S3 **Estimates of Evolutionary Divergence (average) over Sequence Pairs between and within** ***Wolbachia*** **Supergroups.**
(DOC)

## References

[1] Werren JH (1997). Biology of *Wolbachia*.. Annu Rev Entomol.

[2] Aguiar FM, Ilharco FA (2001). Aphids (Homoptera: Aphidoidea) from Madeira Island – New records and corrections.. Boletin sanidade Vegetal, Plagas.

[3] Saridaki A, Bourtzis K (2010). *Wolbachia*: more than just a bug in insects genitals.. Curr Opin Microbiol.

[4] Bandi C, Anderson TJ, Genchi C, Blaxter ML (1998). Phylogeny of *Wolbachia* in filarial nematodes.. Proc Biol Sci.

[5] Werren JH, Windsor DM (2000). *Wolbachia* infection frequencies in insects: evidence of a global equilibrium?. Proc Biol Sci.

[6] Jeyaprakash A, Hoy MA (2000). Long PCR improves *Wolbachia* DNA amplification: wsp sequences found in 76% of sixty-three arthropod species.. Insect Mol Biol.

[7] Hilgenboecker K, Hammerstein P, Schlattmann P, Telschow A, Werren JH (2008). How many species are infected with *Wolbachia*? statistical analysis of current data.. FEMS Microbiol Lett.

[8] Ferri E, Bain O, Barbuto M, Martin C, Lo N (2011). New insights into the evolution of *Wolbachia* infections in filarial nematodes inferred from a large range of screened species.. PLoS One.

[9] Lo N, Paraskevopoulos C, Bourtzis K, O'Neill SL, Werren JH (2007). Taxonomic status of the intracellular bacterium *Wolbachia pipientis*.. Int J Syst Evol Microbiol.

[10] Werren JH, Zhang W, Guo LR (1995). Evolution and phylogeny of *Wolbachia*: reproductive parasites of arthropods.. Proc Biol Sci.

[11] Vandekerckhove TT, Watteyne S, Willems A, Swings JG, Mertens J (1999). Phylogenetic analysis of the 16S rDNA of the cytoplasmic bacterium *Wolbachia* from the novel host Folsomia candida (Hexapoda, Collembola) and its implications for wolbachial taxonomy.. FEMS Microbiol Lett.

[12] Lo N, Casiraghi M, Salati E, Bazzocchi C, Bandi C (2002). How many *wolbachia* supergroups exist?. Mol Biol Evol.

[13] Vaishampayan PA, Dhotre DP, Gupta RP, Lalwani P, Ghate H (2007). Molecular evidence and phylogenetic affiliations of *Wolbachia* in cockroaches.. Mol Phylogenet Evol.

[14] Dunn AK, Stabb EV (2005). Culture-independent characterization of the microbiota of the ant lion *Myrmeleon mobilis* (Neuroptera: Myrmeleontidae).. Appl Environ Microbiol.

[15] Bordenstein S, Rosengaus RB (2005). Discovery of a novel *Wolbachia* super group in Isoptera.. Curr Microbiol.

[16] Haegeman A, Vanholme B, Jacob J, Vandekerckhove TT, Claeys M (2009). An endosymbiotic bacterium in a plant-parasitic nematode: member of a new *Wolbachia* supergroup.. Int J Parasitol.

[17] Ros VI, Fleming VM, Feil EJ, Breeuwer JA (2009). How diverse is the genus *Wolbachia*? Multiple-gene sequencing reveals a putatively new *Wolbachia*supergroup recovered from spider mites (Acari: Tetranychidae).. Appl Environ Microbiol.

[18] Rowley SM, Raven RJ, McGraw EA (2004). *Wolbachia pipientis* in Australian spiders.. Curr Microbiol.

[19] Baldo L, Prendini L, Corthals A, Werren JH (2007). *Wolbachia* are present in southern african scorpions and cluster with supergroup F.. Curr Microbiol.

[20] Baldo L, Dunning Hotopp JC, Jolley KA, Bordenstein SR, Biber SA (2006). Multilocus sequence typing system for the endosymbiont *Wolbachia pipientis*.. Appl Environ Microbiol.

[21] Paraskevopoulos C, Bordenstein SR, Wernegreen JJ, Werren JH, Bourtzis K (2006). Toward a *Wolbachia* multilocus sequence typing system: discrimination of *Wolbachia* strains present in Drosophila species.. Curr Microbiol.

[22] Stouthamer R, Breeuwer JA, Hurst GD (1999). *Wolbachia pipientis*: microbial manipulator of arthropod reproduction.. Annu Rev Microbiol.

[23] Hancock PA, Sinkins SP, Godfray HC (2011). Population dynamic models of the spread of *Wolbachia*.. Am Nat.

[24] Bourtzis K, Dobson SL, Braig HR, O'Neill SL (1998). Rescuing *Wolbachia* have been overlooked.. Nature.

[25] Bourtzis K, Robinson AS, Bourtzis K, Miller T (2006). Insect pest control using *Wolbachia* and/or radiation.. Insect Symbiosis 2.

[26] Bourtzis K (2008). *Wolbachia*-based technologies for insect pest population control.. Adv Exp Med Biol.

[27] Laven H (1967). Eradication of *Culex pipiens fatigans* through cytoplasmic incompatibility.. Nature.

[28] Zabalou S, Riegler M, Theodorakopoulou M, Stauffer C, Savakis C (2004). *Wolbachia*-induced cytoplasmic incompatibility as a means for insect pest population control.. Proc Natl Acad Sci U S A.

[29] Xi Z, Dean JL, Khoo C, Dobson SL (2005). Generation of a novel *Wolbachia* infection in *Aedes albopictus* (Asian tiger mosquito) via embryonic microinjection.. Insect Biochem Mol Biol.

[30] Kambris Z, Cook PE, Phuc HK, Sinkins SP (2009). Immune activation by life-shortening *Wolbachia* and reduced filarial competence in mosquitoes.. Science.

[31] Turley AP, Moreira LA, O'Neill SL, McGraw EA (2009). *Wolbachia* infection reduces blood-feeding success in the dengue fever mosquito, *Aedes aegypti*.. PLoS Negl Trop Dis.

[32] Zabalou S, Apostolaki A, Livadaras I, Franz G, Robinson AS (2009). Incompatible insect technique: incompatible males from a *Ceratitis capitata* genetic sexing strain.. Entomologia Experimentalis et Applicata.

[33] Apostolaki A, Livadaras I, Saridaki A, Chrysargyris A, Savakis C (2011). Transinfection of the olive fruit fly *Bactrocera oleae* with *Wolbachia*: towards a symbiont-based population control strategy.. Journal of Applied Entomology.

[34] McMeniman CJ, Lane RV, Cass BN, Fong AW, Sidhu M (2009). Stable introduction of a life-shortening *Wolbachia* infection into the mosquito *Aedes aegypti*.. Science.

[35] Moreira LA, Iturbe-Ormaetxe I, Jeffery JA, Lu G, Pyke AT (2009). A *Wolbachia* symbiont in *Aedes aegypti* limits infection with dengue, Chikungunya and Plasmodium.. Cell.

[36] Hedges LM, Brownlie JC, O'Neill SL, Johnson KN (2008). *Wolbachia* and virus protection in insects.. Science.

[37] Teixeira L, Ferreira A, Ashburner M (2008). The bacterial symbiont *Wolbachia* induces resistance to RNA viral infections in *Drosophila melanogaster*.. PLoS Biol.

[38] Bian G, Xu Y, Lu P, Xie Y, Xi Z (2010). The endosymbiotic bacterium *Wolbachia* induces resistance to dengue virus in *Aedes aegypti*.. PLoS Pathog.

[39] Cook PE, McGraw EA (2010). *Wolbachia pipientis*: an expanding bag of tricks to explore for disease control.. Trends Parasitol.

[40] Glaser RL, Meola MA (2010). The native *Wolbachia* endosymbionts of *Drosophila melanogaster* and *Culex quinquefasciatus* increase host resistance to West Nile virus infection.. PLoS One.

[41] Dixon AFG, Kindlmann P, Leps J, Holman J (1987). Why There are So Few Species of Aphids, Especially in the Tropics.. The American Naturalist.

[42] Schadler M, Brandl R, Haase J (2007). Antagonistic interactions between plant competition and insect herbivory.. Ecology.

[43] Brault V, Tanguy S, Reinbold C, Le Trionnaire G, Arneodo J (2010). Transcriptomic analysis of intestinal genes following acquisition of pea enation mosaic virus by the pea aphid *Acyrthosiphon pisum*.. J Gen Virol.

[44] Douglas AE (2006). Phloem-sap feeding by animals: Problems and solutions.. Journal of Experimental Botany.

[45] Oliver KM, Degnan PH, Burke GR, Moran NA (2010). Facultative Symbionts in Aphids and the Horizontal Transfer of Ecologically Important Traits.. Annual Review of Entomology.

[46] Douglas AE (1997). Parallels and contrasts between symbiotic bacteria and bacterial-derived organelles: evidence from *Buchnera*, the bacterial symbiont of aphids.. FEMS MicrobiologyEcology.

[47] Baumann P (2005). Biology bacteriocyte-associated endosymbionts of plant sap-sucking insects.. Annu Rev Microbiol.

[48] Moya A, Pereto J, Gil R, Latorre A (2008). Learning how to live together: genomic insights into prokaryote-animal symbioses.. Nat Rev Genet.

[49] Brinza L, Vinuelas J, Cottret L, Calevro F, Rahbe Y (2009). Systemic analysis of the symbiotic function of *Buchnera aphidicola*, the primary endosymbiont of the pea aphid *Acyrthosiphon pisum*.. C R Biol.

[50] Oliver KM, Moran NA, Hunter MS (2005). Variation in resistance to parasitism in aphids is due to symbionts not host genotype.. Proc Natl Acad Sci U S A.

[51] Vorburger C, Gehrer L, Rodriguez P (2010). A strain of the bacterial symbiont *Regiella insecticola* protects aphids against parasitoids.. Biol Lett.

[52] Montllor CB, Maxmen A, Purcell AH (2002). Facultative bacterial endosymbionts benefit pea aphids *Acyrthosiphon pisum* under heat stress.. Ecological Entomology.

[53] Moran NA, Jarvik T (2010). Lateral transfer of genes from fungi underlies carotenoid production in aphids.. Science.

[54] Nikoh N, McCutcheon JP, Kudo T, Miyagishima SY, Moran NA (2010). Bacterial genes in the aphid genome: absence of functional gene transfer from *Buchnera* to its host.. PLoS Genet.

[55] Perez-Brocal V, Gil R, Ramos S, Lamelas A, Postigo M (2006). A small microbial genome: the end of a long symbiotic relationship?. Science.

[56] Gosalbes MJ, Lamelas A, Moya A, Latorre A (2008). The striking case of tryptophan provision in the cedar aphid *Cinara cedri*.. J Bacteriol.

[57] West SA, Cook JM, Werren JH, Godfray HC (1998). *Wolbachia* in two insect host-parasitoid communities.. Mol Ecol.

[58] Tsuchida T, Koga R, Shibao H, Matsumoto T, Fukatsu T (2002). Diversity and geographic distribution of secondary endosymbiotic bacteria in natural populations of the pea aphid, *Acyrthosiphon pisum*.. Mol Ecol.

[59] Nirgianaki A, Banks GK, Frohlich DR, Veneti Z, Braig HR (2003). *Wolbachia* infections of the whitefly *Bemisia tabaci*.. Curr Microbiol.

[60] Gomez-Valero L, Soriano-Navarro M, Perez-Brocal V, Heddi A, Moya A (2004). Coexistence of *Wolbachia* with *Buchnera aphidicola* and a secondary symbiont in the aphid *Cinara cedri*.. J Bacteriol.

[61] Wang Z, Shen ZR, Song Y, Liu HY, Li ZX (2009). Distribution and diversity of *Wolbachia* in different populations of the wheat aphid *Sitobion miscanthi* (Hemiptera: Aphididae) in China.. European Journal of Entomology.

[62] Ortiz-Rivas B, Martinez-Torres D (2010). Combination of molecular data support the existence of three main lineages in the phylogeny of aphids (Hemiptera: Aphididae) and the basal position of the subfamily Lachninae.. Mol Phylogenet Evol.

[63] Zhou W, Rousset F, O'Neil S (1998). Phylogeny and PCR-based classification of *Wolbachia* strains using *wsp* gene sequences.. Proc Biol Sci.

[64] Casiraghi M, Bordenstein SR, Baldo L, Lo N, Beninati T (2005). Phylogeny of *Wolbachia pipientis* based on *gltA*, *groEL* and *ftsZ* gene sequences: clustering of arthropod and nematode symbionts in the F supergroup, and evidence for further diversity in the *Wolbachia* tree.. Microbiology.

[65] Breeuwer JA, Stouthamer R, Barns SM, Pelletier DA, Weisburg WG (1992). Phylogeny of cytoplasmic incompatibility micro-organisms in the parasitoid wasp genus Nasonia (Hymenoptera: Pteromalidae) based on 16S ribosomal DNA sequences.. Insect Mol Biol.

[66] Stouthamer R, Breeuwert JA, Luck RF, Werren JH (1993). Molecular identification of microorganisms associated with parthenogenesis.. Nature.

[67] Burke GR, Normark BB, Favret C, Moran NA (2009). Evolution and diversity of facultative symbionts from the aphid subfamily Lachninae.. Appl Environ Microbiol.

[68] Lamelas A, Gosalbes MJ, Moya A, Latorre A (2011). New Clues about the Evolutionary History of Metabolic Losses in Bacterial Endosymbionts, Provided by the Genome of *Buchnera aphidicola* from the Aphid *Cinara tujafilina*.. Appl Environ Microbiol.

[69] Sinkins SP, Braig HR, O'Neill SL (1995). *Wolbachia* superinfections and the expression of cytoplasmic incompatibility.. Proc Biol Sci.

[70] Dobson SL, Marsland EJ, Veneti Z, Bourtzis K, O'Neill SL (2002). Characterization of *Wolbachia* host cell range via the in vitro establishment of infections.. Appl Environ Microbiol.

[71] Reuter M, Keller L (2003). High levels of multiple *Wolbachia* infection and recombination in the ant *Formica exsecta*.. Mol Biol Evol.

[72] Kikuchi Y, Fukatsu T (2003). Diversity of *Wolbachia* endosymbionts in heteropteran bugs.. Appl Environ Microbiol.

[73] Duron O, Lagnel J, Raymond M, Bourtzis K, Fort P (2005). Transposable element polymorphism of *Wolbachia* in the mosquito *Culex pipiens*: evidence of genetic diversity, superinfection and recombination.. Mol Ecol.

[74] Li ZX, Hu DX, Song Y, Shen ZR (2005). Molecular differentiation of the B biotype from other biotypes of *Bemisia tabaci* (Hemiptera: Aleyrodidae), based on internally transcribed spacer 1 sequences.. European Journal of Entomology.

[75] Li ZX, Lin HZ, Guo XP (2007). Prevalence of *Wolbachia* infection in *Bemisia tabaci*.. Curr Microbiol.

[76] O'Neill SL, Giordano R, Colbert AM, Karr TL, Robertson HM (1992). 16S rRNA phylogenetic analysis of the bacterial endosymbionts associated with cytoplasmic incompatibility in insects.. Proc Natl Acad Sci U S A.

[77] Simoes PM, Mialdea G, Reiss D, Sagot MF, Charlat S (2011). Wolbachia detection: an assessment of standard PCR protocols.. Mol Ecol Resour.

[78] Arthofer W, Riegler M, Schneider D, Krammer M, Miller WJ (2009). Hidden *Wolbachia* diversity in field populations of the European cherry fruit fly, *Rhagoletis cerasi* (Diptera, Tephritidae).. Mol Ecol.

[79] Miller WJ, Ehrman L, Schneider D (2010). Infectious speciation revisited: impact of symbiont-depletion on female fitness and mating behaviour of *Drosophila paulistorum*.. PLoS Pathog.

[80] Arthofer W, Riegler M, Schuler H, Schneider D, Moder K (2011). Allele intersection analysis: a novel tool for multi locus sequence assignment in multiply infected hosts.. PLoS One.

[81] Mouton L, Henri H, Bouletreau M, Vavre F (2003). Strain-specific regulation of intracellular *Wolbachia* density in multiply infected insects.. Mol Ecol.

[82] Kondo N, Shimada M, Fukatsu T (2005). Infection density of *Wolbachia* endosymbiont affected by co-infection and host genotype.. Biol Lett.

[83] Vautrin E, Vavre F (2009). Interactions between vertically transmitted symbionts: cooperation or conflict?. Trends Microbiol.

[84] Kondo N, Nikoh N, Ijichi N, Shimada M, Fukatsu T (2002). Genome fragment of *Wolbachia* endosymbiont transferred to X chromosome of host insect.. Proc Natl Acad Sci U S A.

[85] Fenn K, Conlon C, Jones M, Quail MA, Holroyd NE (2006). Phylogenetic relationships of the *Wolbachia* of nematodes and arthropods.. PLoS Pathog.

[86] Dunning Hotopp JC, Clark ME, Oliveira DC, Foster JM, Fischer P (2007). Widespread lateral gene transfer from intracellular bacteria to multicellular eukaryotes.. Science.

[87] McNulty SN, Foster JM, Mitreva M, Dunning Hotopp JC, Martin J (2010). Endosymbiont DNA in endobacteria-free filarial nematodes indicates ancient horizontal genetic transfer.. PLoS One.

[88] Doudoumis V, Tsiamis G, Wamwiri F, Brelsfoard C, Alam U (2011). Detection and characterization of *Wolbachia* infections in laboratory and natural populations of different species of tse-tse flies (genus *Glossina*).. BMC Microbiology.

[89] IAGC (2010). Genome sequence of the pea aphid *Acyrthosiphon pisum*.. PLoS Biol.

[90] Buchner P (1965). Endosymbiosis of Animals with Plant Microorganisms.

[91] Moran NA, Degnan PH, Santos SR, Dunbar HE, Ochman H (2005). The players in a mutualistic symbiosis: insects, bacteria, viruses, and virulence genes.. Proc Natl Acad Sci U S A.

[92] Degnan PH, Yu Y, Sisneros N, Wing RA, Moran NA (2009a). *Hamiltonella defensa*, genome evolution of protective bacterial endosymbiont from pathogenic ancestors.. Proc Natl Acad Sci U S A.

[93] Degnan PH, Leonardo TE, Cass BN, Hurwitz B, Stern D (2009b). Dynamics of genome evolution in facultative symbionts of aphids.. Environ Microbiol.

[94] Burke GR, Normark BB, Favret C, Moran NA (2009). Evolution and diversity of facultative symbionts from the aphid subfamily Lachninae.. Appl Environ Microbiol.

[95] Lamelas A, Gosalbes MJ, Moya A, Latorre A (2011). New Clues about the Evolutionary History of Metabolic Losses in Bacterial Endosymbionts, Provided by the Genome of *Buchnera aphidicola* from the Aphid *Cinara tujafilina*.. Appl Environ Microbiol.

[96] Hosokawa T, Koga R, Kikuchi Y, Meng XY, Fukatsu T (2010). *Wolbachia* as a bacteriocyte-associated nutritional mutualist.. Proc Natl Acad Sci U S A.

[97] Heie OE (2004). The history of the studies on aphid palaeontology and their bearing on the evolutionary history of aphids.

[98] Aguiar FM, Ilharco FA (2001). Aphids (Homoptera: Aphidoidea) from Madeira Island – New records and corrections.. Boletin sanidade Vegetal, Plagas.

[99] Blackman RL, Eastop VF (1984). Aphids on the world's crops: an identification and information guide.

[100] Blackman RL, Eastop VF (2000). Aphids on the world's crops: an identification and information guide.

[101] Remaudiere G, Remaudiere M (1997). Catalogue des Aphididae du Monde..

[102] Latorre A, Moya A, Ayala FJ (1986). Evolution of mitochondrial DNA in *Drosophila subobscura*.. Proc Natl Acad Sci U S A.

[103] Doyle JJ, Doyle JL (1990). Isolation of plant DNA from fresh tissue.. Focus.

[104] Hanner R, Fugate M (1997). Branchiopod phylogenetic reconstruction from 12S rDNA sequence data.. Journal of Crustacean Biology.

[105] Hartley JL, Bowen H (2003). PEG precipitation for selective removal of small DNA fragments.. Focus.

[106] Reed DW, Fujita Y, Delwiche ME, Blackwelder DB, Sheridan PP (2002). Microbial communities from methane hydrate-bearing deep marine sediments in a forearc basin.. Appl Environ Microbiol.

[107] Edgar RC (2004). MUSCLE: multiple sequence alignment with high accuracy and high throughput.. Nucleic Acids Res.

[108] Thompson JD, Higgins DG, Gibson TJ (1994). CLUSTAL W: improving the sensitivity of progressive multiple sequence alignment through sequence weighting, position-specific gap penalties and weight matrix choice.. Nucleic Acids Res.

[109] Swofford DL (2000). PAUP: phylogenetic analysis using parsimony, 4.0, beta version 4a ed..

[110] Ronquist F, Huelsenbeck JP (2003). MrBayes 3: Bayesian phylogenetic inference under mixed models.. Bioinformatics.

[111] Heath L, van der Walt E, Varsani A, Martin DP (2006). Recombination patterns in aphthoviruses mirror those found in other picornaviruses.. J Virol.

