# Supplementary figures and images for: Detection and Characterization of Wolbachia Infections in Natural Populations of Aphids: Is the Hidden Diversity Fully Unraveled?
Source: PLoS One. 2011 Dec 13;6(12):e28695. doi: 10.1371/journal.pone.0028695 (PMC3236762; doi:10.1371/journal.pone.0028695)

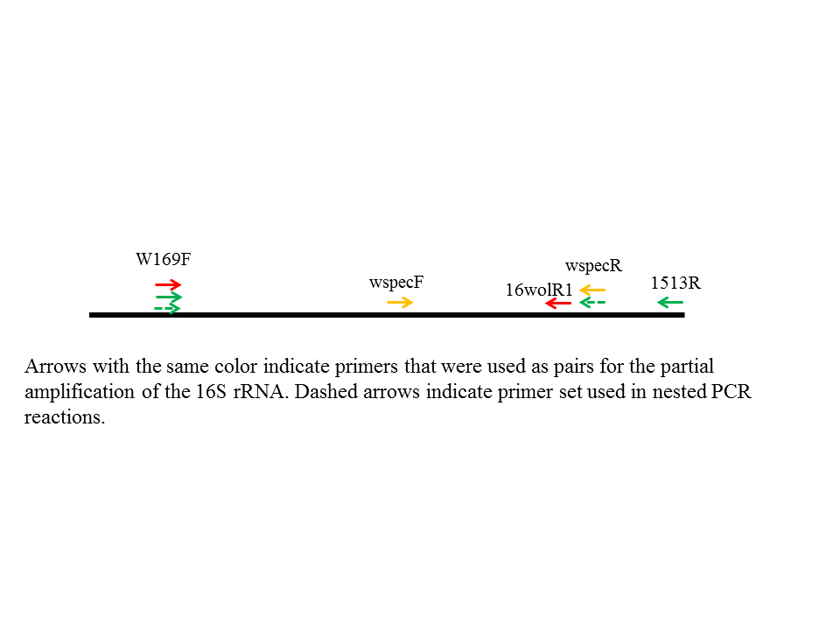

Supplement: Figure S1 — Position of the primers used in this study, relative to the 16S rRNA gene from wMel. (TIF) [file pone.0028695.s001.tif]

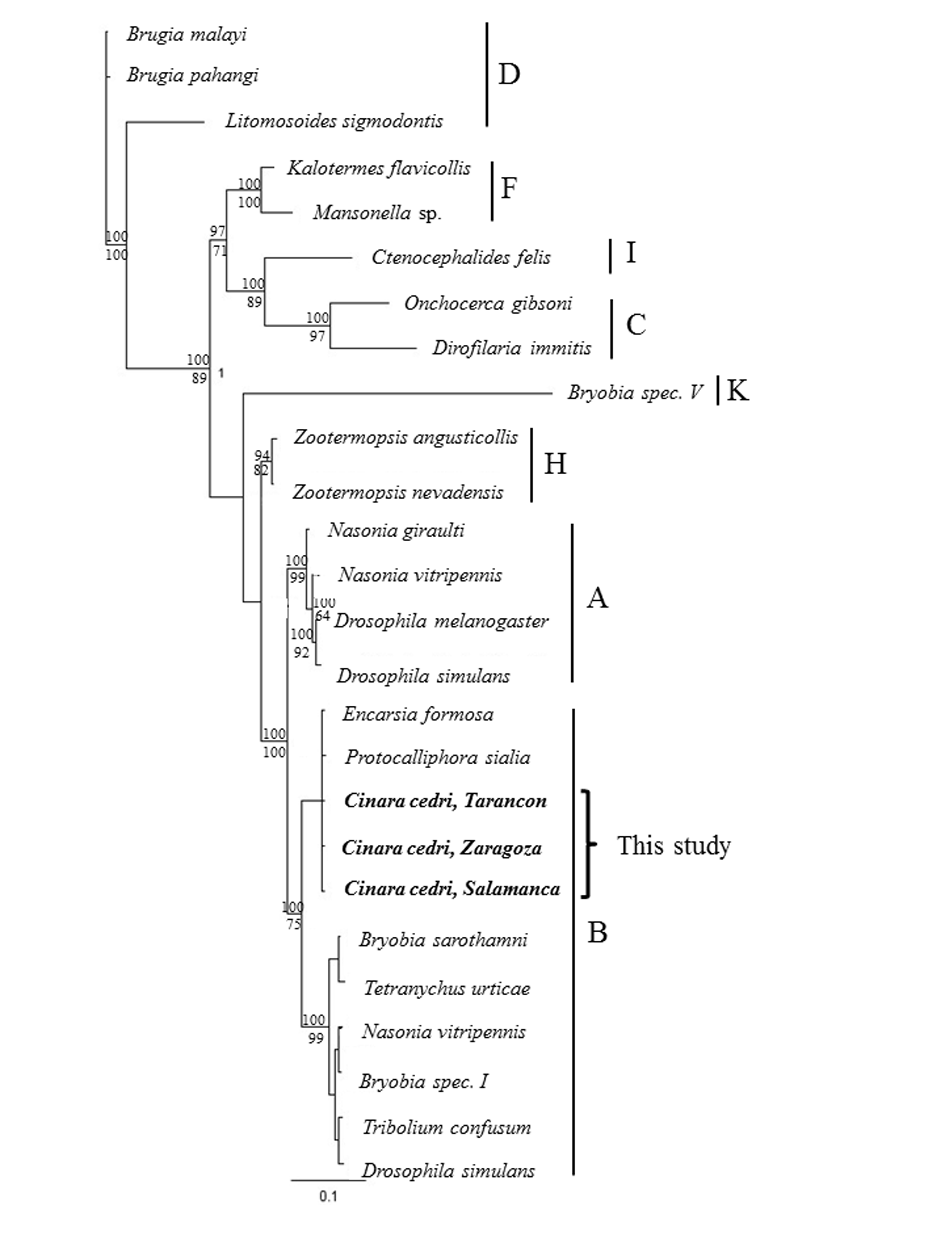

Supplement: Figure S2 — Bayesian inference phylogeny based on gltA data. The three new Wolbachia strains are indicated with bold letters, and the other strains represent supergroups A, B, C, D, F, H, I, and K. Strains are designated with the names of their host species, followed by the collection site and the sample name. Bayesian posterior probabilities (top numbers) and ML bootstrap values based on 100 replicates (bottom numbers) are given. (TIF) [file pone.0028695.s002.tif]

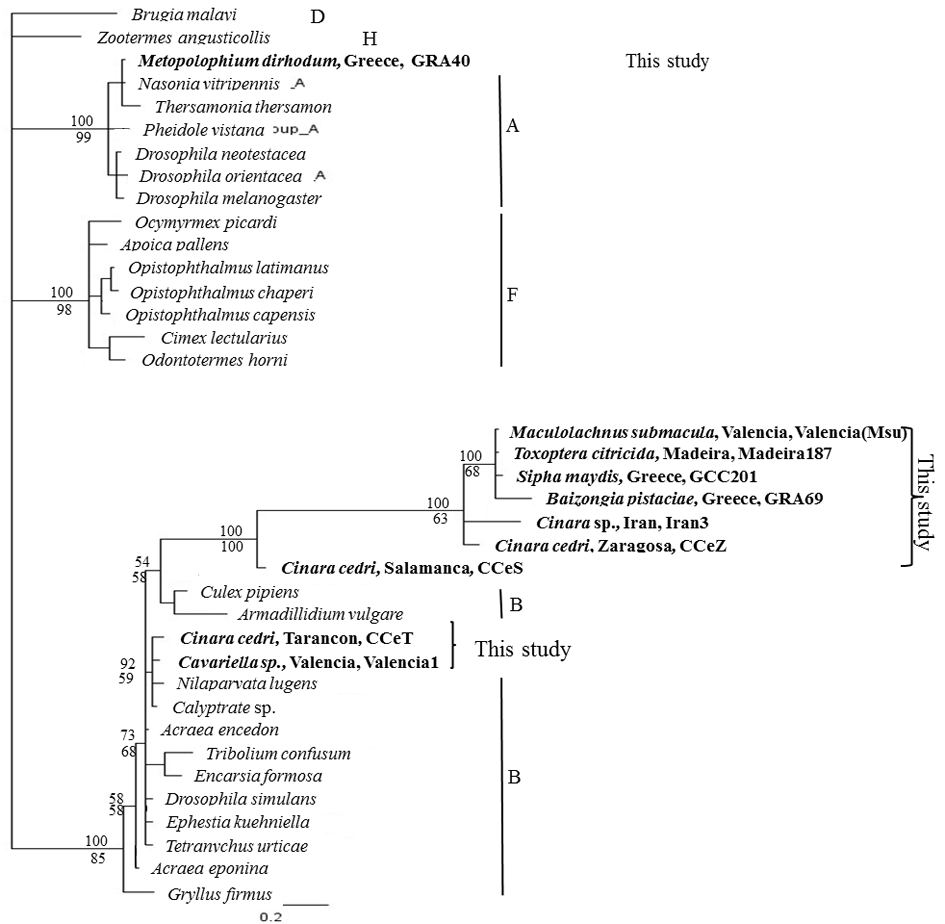

Supplement: Figure S3 — Bayesian inference phylogeny based on gatB data. The 10 new Wolbachia strains are indicated with bold letters, and the other strains represent supergroups A, B, D, F, and H. Strains are designated with the names of their host species, followed by the collection site and the sample name. Bayesian posterior probabilities (top numbers) and ML bootstrap values based on 100 replicates (bottom numbers) are given. (TIF) [file pone.0028695.s003.tif]

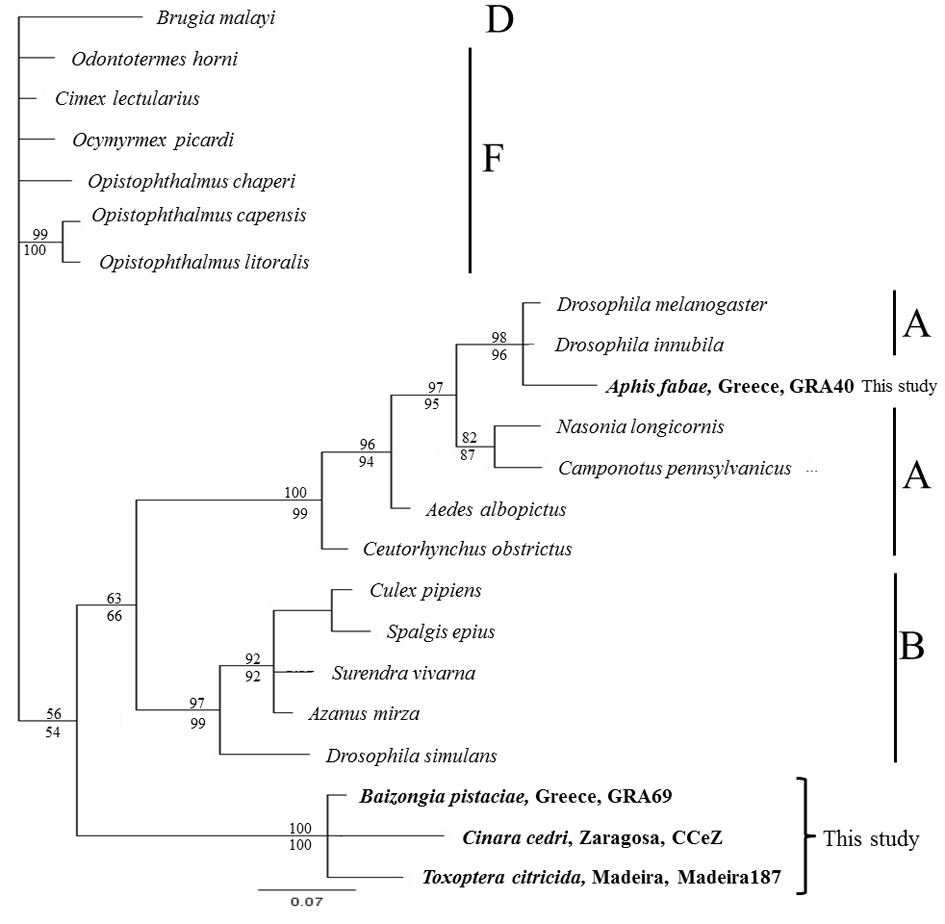

Supplement: Figure S4 — Bayesian inference phylogeny based on fbpA data. The four new Wolbachia strains are indicated with bold letters, and the other strains represent supergroups A, B, D, and F. Strains are designated with the names of their host species, followed by the collection site and the sample name. Bayesian posterior probabilities (top numbers) and ML bootstrap values based on 100 replicates (bottom numbers) are given. (TIF) [file pone.0028695.s004.tif]

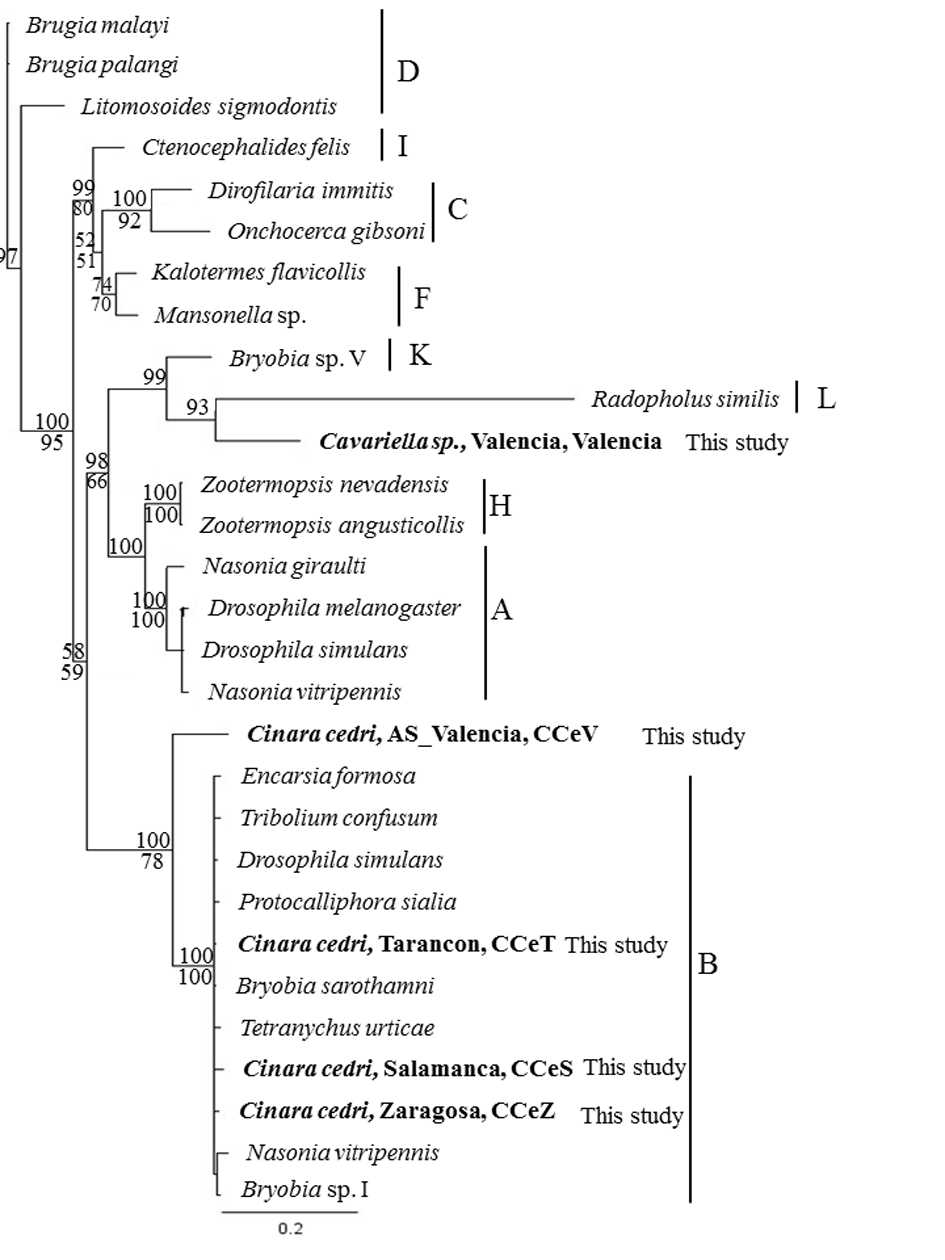

Supplement: Figure S5 — Bayesian inference phylogeny based on groEL data. The five new Wolbachia strains are indicated with bold letters and the other strains represent supergroups A, B, C, D, F, H, I, K, and L. Strains are designated with the names of their host species, followed by the collection site and the sample name. Bayesian posterior probabilities (top numbers) and ML bootstrap values based on 100 replicates (bottom numbers) are given. (TIF) [file pone.0028695.s005.tif]
