# Supplementary material for: Detection and Characterization of Wolbachia Infections in Natural Populations of Aphids: Is the Hidden Diversity Fully Unraveled?
Source: PLoS One. 2011 Dec 13;6(12):e28695. doi: 10.1371/journal.pone.0028695 (PMC3236762; doi:10.1371/journal.pone.0028695)
Supplement: Table S1 — Wolbachia detection of all aphid populations examined in this study, based on 16S rDNA gene sequencing. (DOC) [file pone.0028695.s006.doc]

**Table S1**. *Wolbachia* detection of all the aphid populations examined in this study. (+) indicates presence of *Wolbachia* verified by PCR amplification and partial sequence determination of the 16S rRNA gene, (-) absence of *Wolbachia* based on failure to detect a *Wolbachia-*specific 16S rRNA gene amplification product.

| **Sample** | **Country** | ***Region*** | **Subfamily** | **Tribe** | **Species*** | ***Host plant*** | **Wolbachia presence** |
| --- | --- | --- | --- | --- | --- | --- | --- |
| GRB143 | Greece | Etoloakarnania | Aphidinae | Macrosiphini | *Acyrthosiphon pisum* | *Lathyrus* sp. | - |
| GRC295 | Greece | Etoloakarnania | Aphidinae | Macrosiphini | *Acyrthosiphon pisum* | *Pisum sativum* | - |
| GRB141 | Greece | Etoloakarnania | Aphidinae | Macrosiphini | *Acyrthosiphon lactucae* | *Lactuca serriola* | - |
| GRC219 | Greece | Etoloakarnania | Aphidinae | Macrosiphini | *Acyrthosiphon lactucae* | *Lactuca serriola* | - |
| GRA65 | Greece | Etoloakarnania | Aphidinae | Aphidini | *Aphis balloticola* | *Ballota nigra* | - |
| GRC249 | Greece | Etoloakarnania | Aphidinae | Aphidini | *Aphis balloticola* | *Ballota nigra* | - |
| GRB127 | Greece | Etoloakarnania | Aphidinae | Aphidini | *Aphis espiraecola* | *Citrus aurantium* | - |
| GRC285 | Greece | Etoloakarnania | Aphidinae | Aphidini | *Aphis espiraecola* | *Conyza canadensis* | - |
| GRC286 | Greece | Etoloakarnania | Aphidinae | Aphidini | *Aphis espiraecola* | *Yucca* sp. | - |
| GRA5 | Greece | Etoloakarnania | Aphidinae | Aphidini | *Aphis craccae* | *Vicia* sp. | - |
| GRB137 | Greece | Etoloakarnania | Aphidinae | Aphidini | *Aphis craccae* | *Vicia craccae* | - |
| GRC223 | Greece | Etoloakarnania | Aphidinae | Aphidini | *Aphis craccae* | *Vicia craccae* | - |
| GRB100 | Greece | Etoloakarnania | Aphidinae | Aphidini | *Aphis craccivora* | *Vicia* sp. | - |
| GRC208 | Greece | Etoloakarnania | Aphidinae | Aphidini | *Aphis craccivora* | *Vicia faba* | - |
| GRB155 | Greece | Etoloakarnania | Aphidinae | Aphidini | *Aphis craccivora* | *Melilotus officinalis* | - |
| GRA49 | Greece | Etoloakarnania | Aphidinae | Aphidini | *Αphis craccivora* | *Robinia pseudacacia* | - |
| GRA52 | Greece | Etoloakarnania | Aphidinae | Aphidini | *Aphis cytisorum* | *Spartium junceum* | - |
| GRB102 | Greece | Etoloakarnania | Aphidinae | Aphidini | *Aphis cytisorum* | *Spartium junceum* | - |
| GRA56 | Greece | Etoloakarnania | Aphidinae | Aphidini | *Aphis fabae* | *Foeniculum vulgare* | - |
| GRB136 | Greece | Etoloakarnania | Aphidinae | Aphidini | *Aphis fabae* | *Gallium spurium* | - |
| GRC243 | Greece | Etoloakarnania | Aphidinae | Aphidini | *Aphis fabae* | *Papaver rhoeas* | - |
| GRA4 | Greece | Etoloakarnania | Aphidinae | Aphidini | *Aphis fabae* | *Phaseolus vulgaris* | - |
| GRA80 | Greece | Etoloakarnania | Aphidinae | Aphidini | *Aphis fabae* | *Rumex* sp. | - |
| GRB107 | Greece | Etoloakarnania | Aphidinae | Aphidini | *Aphis fabae* | *Rumex crispus* | - |
| GRA21 | Greece | Etoloakarnania | Aphidinae | Aphidini | *Aphis fabae* | *Solanum nigrum* | - |
| GRB132 | Greece | Etoloakarnania | Aphidinae | Aphidini | *Aphis fabae* | *Solanum nigrum* | - |
| GRC226 | Greece | Etoloakarnania | Aphidinae | Aphidini | *Aphis fabae* | *Solanum nigrum* | - |
| GRA61 | Greece | Etoloakarnania | Aphidinae | Aphidini | *Aphis fabae* | *Tulipa* sp. | - |
| GRB125 | Greece | Etoloakarnania | Aphidinae | Aphidini | *Aphis fabae* | *Vicia faba* | - |
| GRA18 | Greece | Etoloakarnania | Aphidinae | Aphidini | *Aphis gossypii* | *Cucumis sativus* | - |
| GRB131 | Greece | Etoloakarnania | Aphidinae | Aphidini | *Aphis gossypii* | *Gossypium hirsutum* | - |
| GRA13 | Greece | Etoloakarnania | Aphidinae | Aphidini | *Aphis gossypii* | *Hibiscus* sp. | - |
| GRC281 | Greece | Etoloakarnania | Aphidinae | Aphidini | *Aphis gossypii* | *Hibiscus syriacus* | - |
| GRA17 | Greece | Etoloakarnania | Aphidinae | Aphidini | *Aphis hederae* | *Hedera helix* | + |
| GRB140 | Greece | Etoloakarnania | Aphidinae | Aphidini | *Aphis hederae* | *Hedara helix* | - |
| GRC268 | Greece | Etoloakarnania | Aphidinae | Aphidini | *Aphis hederae* | *Hedera helix* | - |
| GRC213 | Greece | Etoloakarnania | Aphidinae | Aphidini | *Aphis illinoisensis* | *Vitis vinifera* | - |
| GRB135 | Greece | Etoloakarnania | Aphidinae | Aphidini | *Aphis intybi* | *Cichorium intybus* | - |
| GRC202 | Greece | Etoloakarnania | Aphidinae | Aphidini | *Aphis intybi* | *Cichorium intybus* | - |
| GRA29 | Greece | Etoloakarnania | Aphidinae | Aphidini | *Aphis nerii* | *Nerium oleander* | - |
| GRB156 | Greece | Etoloakarnania | Aphidinae | Aphidini | *Aphis nerii* | *Nerium oleander* | - |
| GRC224 | Greece | Etoloakarnania | Aphidinae | Aphidini | *Aphis nerii* | *Nerium oleander* | - |
| GRA59 | Greece | Etoloakarnania | Aphidinae | Aphidini | *Aphis pomi* | *Malus domestica* | - |
| GRB103 | Greece | Etoloakarnania | Aphidinae | Aphidini | *Aphis pomi* | *Malus sylvestris* | - |
| GRC289 | Greece | Etoloakarnania | Aphidinae | Aphidini | *Aphis pomi* | *Cotoneaster* sp. | - |
| GRC248 | Greece | Etoloakarnania | Aphidinae | Aphidini | *Aphis parietariae* | *Parietaria judaica* | - |
| GRA82 | Greece | Etoloakarnania | Aphidinae | Aphidini | *Aphis punicae* | *Punica granatum* | - |
| GRB109 | Greece | Etoloakarnania | Aphidinae | Aphidini | *Aphis punicae* | *Punica granatum* | - |
| GRC228 | Greece | Etoloakarnania | Aphidinae | Aphidini | *Aphis punicae* | *Punica granatum* | - |
| GRB158 | Greece | Etoloakarnania | Aphidinae | Aphidini | *Aphis ruborum* | *Rubus fruticosus* | - |
| GRA6 | Greece | Etoloakarnania | Aphidinae | Aphidini | *Aphis ruborum* | *Rubus* sp. | - |
| GRC218 | Greece | Etoloakarnania | Aphidinae | Aphidini | *Aphis ruborum* | *Rubus fruticosus* | - |
| GRA27 | Greece | Etoloakarnania | Aphidinae | Aphidini | *Aphis rumicis* | *Rumex* sp. | - |
| GRC277 | Greece | Etoloakarnania | Aphidinae | Aphidini | *Aphis rumicis* | *Rumex crispus* | - |
| GRA16 | Greece | Etoloakarnania | Aphidinae | Macrosiphini | *Aulacorthum solani* | *Solanum nigrum* | - |
| GRA78 | Greece | Etoloakarnania | Aphidinae | Aphidini | *Aphis spiraecola* | *Fotinia* sp. | - |
| GRA8 | Greece | Etoloakarnania | Aphidinae | Aphidini | *Aphis urticata* | *Urtica dioica* | - |
| GRA44 | Greece | Etoloakarnania | Aphidinae | Aphidini | *Aphis umbrella* | *Malva* sp. | - |
| GRB106 | Greece | Etoloakarnania | Aphidinae | Aphidini | *Aphis umbrella* | *Malva parviflora* | - |
| GRA8 | Greece | Etoloakarnania | Aphidinae | Aphidini | *Aphis urticata* | *Urtica dioica* | - |
| GRA66 | Greece | Etoloakarnania | Aphidinae | Aphidini | *Aphis sp.* | *Vitis* sp. | - |
| GRA71 | Greece | Etoloakarnania | Aphidinae | Aphidini | *Aphis gossypii* | *Vinca major* | - |
| GRA72 | Greece | Etoloakarnania | Aphidinae | Aphidini | *Aphis spiraephaga* | *Spiraea* sp. | - |
| GRC299 | Greece | Etoloakarnania | Aphidinae | Macrosiphini | *Aulacorthum solani* | *Solanum nigrum* | - |
| GRA28 | Greece | Etoloakarnania | Aphidinae | Macrosiphini | *Brachycaudus cardui* | *Cirsium* sp. | - |
| GRB139 | Greece | Etoloakarnania | Aphidinae | Macrosiphini | *Brachycaudus cardui* | *Cirsium arvense* | - |
| GRC232 | Greece | Etoloakarnania | Aphidinae | Macrosiphini | *Brachycaudus cardui* | *Cynara scolymus* | - |
| GRA43 | Greece | Etoloakarnania | Aphidinae | Macrosiphini | *Brachycaudus helichrysi* | *Prunus domestica* | - |
| GRC287 | Greece | Etoloakarnania | Aphidinae | Macrosiphini | *Brachycaudus helichrysi* | *Senecio vulgaris* | - |
| GRA41 | Greece | Etoloakarnania | Aphidinae | Macrosiphini | *Brevicoryne brassicae* | *Brassica oleracea* | - |
| GRB124 | Greece | Etoloakarnania | Aphidinae | Macrosiphini | *Brevicoryne brassicae* | *Brassica oleraceus* | - |
| GRC247 | Greece | Etoloakarnania | Aphidinae | Macrosiphini | *Brevicoryne brassicae* | *Brassica oleracea* | - |
| GRA1 | Greece | Etoloakarnania | Aphidinae | Macrosiphini | *Brachycaudus persicae* | *Prunus armeniaca* | - |
| GRA11 | Greece | Etoloakarnania | Aphidinae | Macrosiphini | *Brachycaudus persicae* | *Prunus persica* | - |
| GRC231 | Greece | Etoloakarnania | Aphidinae | Macrosiphini | *Brachycaudus persicae* | *Prunus persica* | - |
| GRC205 | Greece | Etoloakarnania | Aphidinae | Macrosiphini | *Brachycaudus tragopogonis* | *Tragopogon* sp. | - |
| GRA25 | Greece | Etoloakarnania | Aphidinae | Macrosiphini | *Brachycaudus sp.* | *Tragopogon* sp. | - |
| GRA12 | Greece | Etoloakarnania | Aphidinae | Macrosiphini | *Capitophorus elaeaegni* | *Cirsium* sp. | - |
| GRB129 | Greece | Etoloakarnania | Aphidinae | Macrosiphini | *Capitophorus elaeaegni* | *Cirsium arvense* | - |
| GRC252 | Greece | Etoloakarnania | Aphidinae | Macrosiphini | *Capitophorus elaeaegni* | *Eleagnus* sp. | - |
| GRA7 | Greece | Etoloakarnania | Aphidinae | Macrosiphini | *Cavariella aegopodii* | *Daucus carota* | - |
| GRC274 | Greece | Etoloakarnania | Aphidinae | Macrosiphini | *Cavariella aegopodii* | *Daucus carota* | - |
| GRB116 | Greece | Etoloakarnania | Aphidinae | Macrosiphini | *Coloradoa rufomaculata* | *Chrysanthemum* sp. | - |
| GRC235 | Greece | Etoloakarnania | Aphidinae | Macrosiphini | *Dysaphis apiifolia* | *Apium graveolens* | - |
| GRA37 | Greece | Etoloakarnania | Aphidinae | Macrosiphini | *Dysaphis plantaginea* | *Malus domestica* | - |
| GRC284 | Greece | Etoloakarnania | Aphidinae | Macrosiphini | *Dysaphis plantaginea* | *Malus sylvestris* | - |
| GRA30 | Greece | Etoloakarnania | Aphidinae | Macrosiphini | *Dysaphis pyri* | *Pyrus communis* | - |
| GRC234 | Greece | Etoloakarnania | Aphidinae | Macrosiphini | *Dysaphis pyri* | *Pyrus communis* | - |
| GRA85 | Greece | Etoloakarnania | Aphidinae | Macrosiphini | *Hayhurstia atriplicis* | *Chenopodium album* | - |
| GRB133 | Greece | Etoloakarnania | Aphidinae | Macrosiphini | *Hayhurstia atriplicis* | *Chenopodium album* | - |
| GRC214 | Greece | Etoloakarnania | Aphidinae | Macrosiphini | *Hayhurstia atriplicis* | *Chenopodium album* | - |
| GRC203 | Greece | Etoloakarnania | Aphidinae | Macrosiphini | *Hyadaphis coriandri* | *Apium graveolens* | - |
| GRC292 | Greece | Etoloakarnania | Aphidinae | Macrosiphini | *Hyadaphis passerinii* | *Lonicera* sp. | - |
| GRC297 | Greece | Etoloakarnania | Aphidinae | Aphidini | *Hyalopterus* sp. | *Arundo* sp. | - |
| GRA84 | Greece | Etoloakarnania | Aphidinae | Aphidini | *Hyalopterus* sp*.* | *Phragmites* sp. | - |
| GRA10 | Greece | Etoloakarnania | Aphidinae | Aphidini | *Hyalopterus amygdali* | *Prunus persica* | - |
| GRB126 | Greece | Etoloakarnania | Aphidinae | Aphidini | *Hyalopterus pruni* | *Prunus domestica* | - |
| GRC298 | Greece | Etoloakarnania | Aphidinae | Aphidini | *Hyalopterus* sp. | *Prunus* sp. | - |
| GRA42 | Greece | Etoloakarnania | Aphidinae | Macrosiphini | *Hyperomyzus lactucae* | *Sonchus oleraceus* | - |
| GRB112 | Greece | Etoloakarnania | Aphidinae | Macrosiphini | *Hyperomyzus lactucae* | *Sonchus oleraceus* | - |
| GRC276 | Greece | Etoloakarnania | Aphidinae | Macrosiphini | *Hyperomyzus lactucae* | *Sonchus oleraceus* | - |
| GRB122 | Greece | Etoloakarnania | Aphidinae | Macrosiphini | *Hyperomyzus pallidus* | *Sonchus arvensis* | - |
| GRA55 | Greece | Etoloakarnania | Aphidinae | Macrosiphini | *Lipaphis erysimi* | *Sinapis* sp*.* | - |
| GRB117 | Greece | Etoloakarnania | Aphidinae | Macrosiphini | *Lipaphis erysimi* | *Sinapis* sp. | - |
| GRA73 | Greece | Etoloakarnania | Aphidinae | Macrosiphini | *Macrosiphum euphorbiae* | *Citrus aurantium* | - |
| GRA60 | Greece | Etoloakarnania | Aphidinae | Macrosiphini | *Macrosiphum euphorbiae* | *Conyza* sp. | - |
| GRB134 | Greece | Etoloakarnania | Aphidinae | Macrosiphini | *Macrosiphum euphorbiae* | *Petunia hybrida* | - |
| GRA14 | Greece | Etoloakarnania | Aphidinae | Macrosiphini | *Macrosiphum euphorbiae* | *Solanum* sp. | - |
| GRA58 | Greece | Etoloakarnania | Aphidinae | Macrosiphini | *Macrosiphum euphorbiae* | *Tulipa* sp. | - |
| GRA19 | Greece | Etoloakarnania | Aphidinae | Macrosiphini | *Macrosiphum rosae* | *Rosa* sp. | - |
| GRB128 | Greece | Etoloakarnania | Aphidinae | Macrosiphini | *Macrosiphum rosae* | *Rosa* sp*.* | - |
| GRC267 | Greece | Etoloakarnania | Aphidinae | Macrosiphini | *Macrosiphum rosae* | *Rosa* sp. | - |
| GRA81 | Greece | Etoloakarnania | Aphidinae | Macrosiphini | *Macrosiphoniella sanborni* | *Chrysanthemum* sp. | - |
| GRC264 | Greece | Etoloakarnania | Aphidinae | Macrosiphini | *Macrosiphoniella sanborni* | *Chrysanthemum* sp. | - |
| GRA15 | Greece | Etoloakarnania | Aphidinae | Macrosiphini | *Megoura viciae* | *Vicia faba* | - |
| GRB119 | Greece | Etoloakarnania | Aphidinae | Macrosiphini | *Megoura viciae* | *Vicia faba* | - |
| GRA39 | Greece | Etoloakarnania | Aphidinae | Aphidini | *Melanaphis pyraria* | *Pyrus communis* | - |
| GRA40 | Greece | Etoloakarnania | Aphidinae | Macrosiphini | *Metopolophium dirhodum* | *Triticum aestivum* | - |
| GRB118 | Greece | Etoloakarnania | Aphidinae | Macrosiphini | *Metopolophium dirhodum* | *Triticum aestivum* | - |
| GRC278 | Greece | Etoloakarnania | Aphidinae | Macrosiphini | *Metopolophium dirhodum* | *Triticum aestivum* | - |
| GRA48 | Greece | Etoloakarnania | Aphidinae | Macrosiphini | *Myzus cerasi* | *Prunus avium* | - |
| GRB152 | Greece | Etoloakarnania | Aphidinae | Macrosiphini | *Myzus cerasi* | *Prunus cerasus* | - |
| GRC215 | Greece | Etoloakarnania | Aphidinae | Macrosiphini | *Myzus cerasi* | *Prunus avium* | - |
| GRA63 | Greece | Etoloakarnania | Aphidinae | Macrosiphini | *Myzus persicae* | *Prunus persica* | - |
| GRC239 | Greece | Etoloakarnania | Aphidinae | Macrosiphini | *Myzus persicae* | *Prunus persica* | - |
| GRA57 | Greece | Etoloakarnania | Aphidinae | Macrosiphini | *Myzus persicae* | *Raphanus raphanistrum* | - |
| GRB101 | Greece | Etoloakarnania | Aphidinae | Macrosiphini | *Myzus persicae* | *Sinapis arvensis* | - |
| GRA62 | Greece | Etoloakarnania | Aphidinae | Macrosiphini | *Myzus persicae* | *Tulipa spp.* | - |
| GRB121 | Greece | Etoloakarnania | Aphidinae | Macrosiphini | *Myzus varians* | *Prunus insititia* | - |
| GRA2 | Greece | Etoloakarnania | Aphidinae | Macrosiphini | *Myzus varians* | *Prunus persica* | - |
| GRA20 | Greece | Etoloakarnania | Aphidinae | Macrosiphini | *Ovatus crataegarius* | *Cydonia oblonga* | - |
| GRA26 | Greece | Etoloakarnania | Aphidinae | Macrosiphini | *Pleotrichophorus chrysanthemi* | *Chrysanthemum* sp. | - |
| GRA3 | Greece | Etoloakarnania | Aphidinae | Macrosiphini | *Phorodon humuli* | *Prunus* sp. | - |
| GRC209 | Greece | Etoloakarnania | Aphidinae | Macrosiphini | *Phorodon humuli* | *Prunus ceracifera* | - |
| GRC266 | Greece | Etoloakarnania | Aphidinae | Aphidini | *Rhopalosiphum maidis* | *Zea mays* | - |
| GRA31 | Greece | Etoloakarnania | Aphidinae | Aphidini | *Rhopalosiphum maidis* | *Sorghum halepense* | - |
| GRB138 | Greece | Etoloakarnania | Aphidinae | Aphidini | *Rhopalosiphum maidis* | *Sorghum halepense* | - |
| GRA33 | Greece | Etoloakarnania | Aphidinae | Aphidini | *Rhopalosiphum numphaeae* | *Prunus* sp. | - |
| GRC275 | Greece | Etoloakarnania | Aphidinae | Aphidini | *Rhopalosiphum nymphaeae* | *Prunus domestica* | - |
| GRA9 | Greece | Etoloakarnania | Aphidinae | Aphidini | *Rhopalosiphum padi* | *Triticum aestivum* | - |
| GRC279 | Greece | Etoloakarnania | Aphidinae | Aphidini | *Rhopalosiphum padi* | *Triticum aestivum* | - |
| GRA70 | Greece | Etoloakarnania | Aphidinae | Aphidini | *Schizaphis graminum* | *Sorghum halepense* | - |
| GRB108 | Greece | Etoloakarnania | Aphidinae | Macrosiphini | *Semiaphis dauci* | *Daucus carota* | - |
| GRA23 | Greece | Etoloakarnania | Aphidinae | Macrosiphini | *Sitobion avenae* | *Triticum aestivum* | - |
| GRC216 | Greece | Etoloakarnania | Aphidinae | Macrosiphini | *Sitobion avenae* | *Triticum aestivum* | - |
| GRA35 | Greece | Etoloakarnania | Aphidinae | Macrosiphini | *Sitobion fragariae* | *Bromus* sp. | - |
| GRB105 | Greece | Etoloakarnania | Aphidinae | Macrosiphini | *Sitobion fragariae* | *Bromus sterilis* | - |
| GRC207 | Greece | Etoloakarnania | Aphidinae | Macrosiphini | *Sitobion fragariae* | *Hordeum murinum* | - |
| GRA50 | Greece | Etoloakarnania | Aphidinae | Macrosiphini | *Staticobium limonii* | *Limonium* sp. | - |
| GRB147 | Greece | Etoloakarnania | Aphidinae | Macrosiphini | *Staticobium limonii* | *Limonium* sp. | - |
| GRA32 | Greece | Etoloakarnania | Aphidinae | Aphidini | *Toxoptera aurantii* | *Citrus* sp. | - |
| GRB130 | Greece | Etoloakarnania | Aphidinae | Aphidini | *Toxoptera aurantii* | *Citrus aurantium* | - |
| GRC283 | Greece | Etoloakarnania | Aphidinae | Aphidini | *Toxoptera aurantii* | *Citrus cinensis* | - |
| GRB123 | Greece | Etoloakarnania | Aphidinae | Macrosiphini | *Uroleucon chondrillae* | *Chondrilla juncea* | - |
| GRC262 | Greece | Etoloakarnania | Aphidinae | Macrosiphini | *Uroleucon cichorii* | *Cichorium intybus* | - |
| GRA77 | Greece | Etoloakarnania | Aphidinae | Macrosiphini | *Uroleucon cichorii* | *Citrus* sp. | - |
| GRA22 | Greece | Etoloakarnania | Aphidinae | Macrosiphini | *Uroleucon sonchi* | *Cichorium* sp. | - |
| GRA38 | Greece | Etoloakarnania | Aphidinae | Macrosiphini | *Uroleucon spp.* | *Sonchus oleraceus* | - |
| GRB120 | Greece | Etoloakarnania | Aphidinae | Macrosiphini | *Uroleucon sonchi* | *Sonchus oleraceus* | - |
| GRC288 | Greece | Etoloakarnania | Aphidinae | Macrosiphini | *Uroleucon sonchi* | *Sonchus oleraceus* | - |
| GRB150 | Greece | Etoloakarnania | Aphidinae | Macrosiphini | *Uromelan aeneus* | *Cirsium* sp. | - |
| 10Ir16 | Iran | Astara | Aphidinae | Aphidini | *Aphis nerii* | *Nerium orlander* | - |
| 10Ir24 | Iran | Tehran | Aphidinae | Aphidini | *Aphis nerii* | *Nerium orlander* | - |
| 09Ir1 | Iran | Tehran | Aphidinae | Aphidini | *Aphis nerii* | *Nerium oleander* | - |
| 10Az12 | Portugal | Terceira (Azores) | Aphidinae | Aphidini | *Aphis affinis* | *Rubia* sp*.* | - |
| 10Az25 | Portugal | Terceira (Azores) | Aphidinae | Aphidini | *Aphis craccivora* | *Arundo donax* | - |
| 10Az24 | Portugal | Terceira (Azores) | Aphidinae | Aphidini | *Aphis craccivora* | *Umbilicus* sp*.* | - |
| 10Az26 | Portugal | Terceira (Azores) | Aphidinae | Aphidini | *Aphis cytisorum* | *Tamarix* sp*.* | - |
| 10Az15 | Portugal | Terceira (Azores) | Aphidinae | Aphidini | *Aphis fabae* | *Acanthus mollis* | - |
| 10Az39 | Portugal | Terceira (Azores) | Aphidinae | Aphidini | *Aphis fabae* | *Arrhenatherum* sp*.* | - |
| 10Az19 | Portugal | Terceira (Azores) | Aphidinae | Aphidini | *Aphis fabae* | *Erica* sp*.* | - |
| 10Az6 | Portugal | Terceira (Azores) | Aphidinae | Aphidini | *Aphis fabae* | *Hedera* sp*.* | - |
| 10Az38 | Portugal | Terceira (Azores) | Aphidinae | Aphidini | *Aphis fabae* | NI | - |
| 10Az9 | Portugal | Terceira (Azores) | Aphidinae | Aphidini | *Aphis gossypii* | *Mentha* sp*.* | - |
| 10Az8 | Portugal | Terceira (Azores) | Aphidinae | Aphidini | *Aphis gossypii* | *Solanum nigrum* | - |
| 10Az22 | Portugal | Terceira (Azores) | Aphidinae | Aphidini | *Aphis gossypii* | *Vicia* sp*.* | - |
| 10Az29 | Portugal | Terceira (Azores) | Aphidinae | Aphidini | *Aphis gossypii* | NI | - |
| 10Az30 | Portugal | Terceira (Azores) | Aphidinae | Aphidini | *Aphis nerii* | NI | - |
| 10Az23 | Portugal | Terceira (Azores) | Aphidinae | Aphidini | *Aphis ruborum* | *Spartium junceum* | - |
| 10Az27 | Portugal | Terceira (Azores) | Aphidinae | Aphidini | *Aphis sedi* | *Nerium oleander* | - |
| 10Az14 | Portugal | Terceira (Azores) | Aphidinae | Aphidini | *Aphis sp.* | NI | - |
| 10Az11 | Portugal | Terceira (Azores) | Aphidinae | Aphidini | *Aphis solanella* | *Kniphofia* sp*.* | - |
| 10Az40 | Portugal | Terceira (Azores) | Aphidinae | Aphidini | *Aphis* sp. | *Agapanthus* sp*.* | - |
| 10Az21 | Portugal | Terceira (Azores) | Aphidinae | Aphidini | *Aphis sp.* | *Lotus* sp*.* | - |
| 10Az16 | Portugal | Terceira (Azores) | Aphidinae | Aphidini | *Aphis sp.* | *Nerium oleander* | + |
| 10Az10 | Portugal | Terceira (Azores) | Aphidinae | Aphidini | *Aphis sp.* | *Strelitzia* sp*.* | + |
| 10Az36 | Portugal | Terceira (Azores) | Aphidinae | Aphidini | *Aphis* sp. | *Tropaeolum majus* | - |
| 10Az28 | Portugal | Terceira (Azores) | Aphidinae | Aphidini | *Melanaphis donacis* | NI | - |
| 10Az32 | Portugal | Terceira (Azores) | Aphidinae | Macrosiphini | *Myzus ornatus* | NI | - |
| 10Az5 | Portugal | Terceira (Azores) | Aphidinae | Aphidini | *Rhopalosiphum padi* | *Annona cherimola* | - |
| 10Az13 | Portugal | Terceira (Azores) | Aphidinae | Aphidini | *Rhopalosiphum padi* | NI | - |
| 10Az2 | Portugal | Terceira (Azores) | Aphidinae | Aphidini | *Sitobion fragariae* | *Arrhenatherum* sp*.* | - |
| 10Az3 | Portugal | Terceira (Azores) | Aphidinae | Aphidini | *Toxoptera aurantii* | *Agapanthus* sp*.* | + |
| 10Az7 | Portugal | Terceira (Azores) | Aphidinae | Aphidini | *Toxoptera aurantii* | *Rumex s* sp*.* | - |
| 10Az17 | Portugal | Terceira (Azores) | Aphidinae | Aphidini | *Toxoptera aurantii* | NI | - |
| 10Az20 | Portugal | Terceira (Azores) | Aphidinae | Macrosiphini | *Uroleucon sonchi* | *Sonchus* sp*.* | - |
| 10Md 155 | Portugal | Madeira | Aphidinae | Macrosiphini | *Acyrthosiphon malvae* | *Geranium purpureum* | - |
| 10Md 94 | Portugal | Madeira | Aphidinae | Macrosiphini | *Acyrthosiphon malvae* | *Geranium* sp*.* | - |
| 10Md 104 | Portugal | Madeira | Aphidinae | Macrosiphini | *Acyrtosiphum* sp. | NI | - |
| 10Md 135 | Portugal | Madeira | Aphidinae | Macrosiphini | *Amphorophora rubi* | *Rubus* sp*.* | - |
| 10Md 168 | Portugal | Madeira | Aphidinae | Aphidini | *Aphis craccivora* | *Bituminaria bituminosa* | - |
| 10Md 191 | Portugal | Madeira | Aphidinae | Aphidini | *Aphis craccivora* | *Cytisus scoparius* | - |
| 10Md 87 | Portugal | Madeira | Aphidinae | Aphidini | *Aphis craccivora* | *Medicago* sp*.* | - |
| 09Md 46 | Portugal | Madeira | Aphidinae | Aphidini | *Aphis craccivora* | NI | - |
| 10Md 141 | Portugal | Madeira | Aphidinae | Aphidini | *Aphis fabae* | *Achyranthes sicula* | - |
| 09Md 6 | Portugal | Madeira | Aphidinae | Aphidini | *Aphis fabae* | *Beta* sp. | - |
| 09Md 12 | Portugal | Madeira | Aphidinae | Aphidini | *Aphis fabae* | *Bidens pilosa* | - |
| 10Md 92 | Portugal | Madeira | Aphidinae | Aphidini | *Aphis fabae* | *Bidens pilosa* | - |
| 10Md 116 | Portugal | Madeira | Aphidinae | Aphidini | *Aphis fabae* | *Centaurium* sp*.* | - |
| 10Md 129 | Portugal | Madeira | Aphidinae | Aphidini | *Aphis fabae* | *Cholophytum colosum* | - |
| 10Md 183 | Portugal | Madeira | Aphidinae | Aphidini | *Aphis fabae* | *Crocosmia crocosmiiflora* | - |
| 10Md 182 | Portugal | Madeira | Aphidinae | Aphidini | *Aphis fabae* | *Digitalis purpurea* | - |
| 10Md 154 | Portugal | Madeira | Aphidinae | Aphidini | *Aphis fabae* | *Erica* sp*.* | - |
| 10Md 122 | Portugal | Madeira | Aphidinae | Aphidini | *Aphis fabae* | *Ficus carica* | - |
| 10Md 146 | Portugal | Madeira | Aphidinae | Aphidini | *Aphis fabae* | *Galactites tomentosa* | - |
| 09Md 2 | Portugal | Madeira | Aphidinae | Aphidini | *Aphis fabae* | *Galium aparine* | - |
| 09Md 33 | Portugal | Madeira | Aphidinae | Aphidini | *Aphis fabae* | *Hebe* sp. | - |
| 09Md 30 | Portugal | Madeira | Aphidinae | Aphidini | *Aphis fabae* | *Hibiscus* sp. | - |
| 10Md 128 | Portugal | Madeira | Aphidinae | Aphidini | *Aphis fabae* | *Hibiscus rosa-sinensis* | - |
| 10Md 136 | Portugal | Madeira | Aphidinae | Aphidini | *Aphis fabae* | *Hordeum* sp*.* | - |
| 09Md 56 | Portugal | Madeira | Aphidinae | Aphidini | *Aphis fabae* | *Jacaranda mimosifolia* | - |
| 10Md 179 | Portugal | Madeira | Aphidinae | Aphidini | *Aphis fabae* | *Phaseolus vulgaris* | - |
| 09Md 25 | Portugal | Madeira | Aphidinae | Aphidini | *Aphis fabae* | *Pittosporum tobira* | - |
| 10Md 153 | Portugal | Madeira | Aphidinae | Aphidini | *Aphis fabae* | *Phyllis nobla* | - |
| 10Md 158 | Portugal | Madeira | Aphidinae | Aphidini | *Aphis fabae* | *Pteridium aquilinum* | - |
| 10Md 115 | Portugal | Madeira | Aphidinae | Aphidini | *Aphis fabae* | *Rubia fruticosa* | - |
| 10Md 150 | Portugal | Madeira | Aphidinae | Aphidini | *Aphis fabae* | *Rumex* sp | - |
| 09Md 20 | Portugal | Madeira | Aphidinae | Aphidini | *Aphis fabae* | *Tropaeolum majus* | - |
| 10Md 134 | Portugal | Madeira | Aphidinae | Aphidini | *Aphis fabae* | *Tropaeolum majus* | - |
| 09Md 19 | Portugal | Madeira | Aphidinae | Aphidini | *Aphis fabae* | *Vicia faba* | - |
| 10Md 100 | Portugal | Madeira | Aphidinae | Aphidini | *Aphis fabae* | *Vicia faba* | - |
| 10Md 164 | Portugal | Madeira | Aphidinae | Aphidini | *Aphis fabae* | *Vitis sp.* | - |
| 09Md 3 | Portugal | Madeira | Aphidinae | Aphidini | *Aphis fabae* | *Vitis* sp. | - |
| 10Md 111 | Portugal | Madeira | Aphidinae | Aphidini | *Aphis fabae* | *Wahlenbergia lobelioides* subsp. *lobelioides* | - |
| 09Md 35 | Portugal | Madeira | Aphidinae | Aphidini | *Aphis fabae* | NI | - |
| 10Md 145 | Portugal | Madeira | Aphidinae | Aphidini | *Aphis fabae* | NI | - |
| 10Md 185 | Portugal | Madeira | Aphidinae | Aphidini | *Aphis fabae* | NI | - |
| 10Md 186 | Portugal | Madeira | Aphidinae | Aphidini | *Aphis fabae* | NI | - |
| 09Md 22 | Portugal | Madeira | Aphidinae | Aphidini | *Aphis farinosa* | *Salix babylonica* | - |
| 09Md 21 | Portugal | Madeira | Aphidinae | Aphidini | *Aphis farinosa* | *Salix canariensis* | - |
| 10Md 107 | Portugal | Madeira | Aphidinae | Aphidini | *Aphis farinosa* | *Salix canariensis* | - |
| 10Md 151 | Portugal | Madeira | Aphidinae | Aphidini | *Aphis* *gossypii* | *Aichryson divaricatum* | - |
| 09Md 57 | Portugal | Madeira | Aphidinae | Aphidini | *Aphis gossypii* | *Cassia didymobotrya* | - |
| 10Md 184 | Portugal | Madeira | Aphidinae | Aphidini | *Aphis gossypii* | *Cassia didymobotrya* | - |
| 09Md 53 | Portugal | Madeira | Aphidinae | Aphidini | *Aphis gossypii* | *Clethra arborea* | - |
| 09Md 67 | Portugal | Madeira | Aphidinae | Aphidini | *Aphis gossypii* | *Eriobotrya japonica* | - |
| 10Md 142 | Portugal | Madeira | Aphidinae | Aphidini | *Aphis gossypii* | *Euphorbia piscatoria* | - |
| 09Md 41 | Portugal | Madeira | Aphidinae | Aphidini | *Aphis gossypii* | *Hibiscus rosa-sinensis* | - |
| 09Md 31 | Portugal | Madeira | Aphidinae | Aphidini | *Aphis gossypii* | *Hibiscus* sp. | - |
| 11Md 200 | Portugal | Madeira | Aphidinae | Aphidini | *Aphis gossypii* | *Hibiscus* sp. | - |
| 10Md 114 | Portugal | Madeira | Aphidinae | Aphidini | *Aphis gossypii* | *Medicago* sp. | - |
| 10Md 132 | Portugal | Madeira | Aphidinae | Aphidini | *Aphis gossypii* | *Solanum mauritianum* | - |
| 10Md 88 | Portugal | Madeira | Aphidinae | Aphidini | *Aphis gossypii* | *Vicia sativa* | - |
| 09Md 47 | Portugal | Madeira | Aphidinae | Aphidini | *Aphis gossypii* | NI | - |
| 10Md 90 | Portugal | Madeira | Aphidinae | Aphidini | *Aphis* *gossypii* | NI | - |
| 10Md 101 | Portugal | Madeira | Aphidinae | Aphidini | *Aphis idaei* | *Rubus* sp. | - |
| 10Md 133 | Portugal | Madeira | Aphidinae | Aphidini | *Aphis nasturtii* | *Lavatera critica* | - |
| 09Md 32 | Portugal | Madeira | Aphidinae | Aphidini | *Aphis nerii* | *Asclepias curassavica* | - |
| 09Md 71 | Portugal | Madeira | Aphidinae | Aphidini | *Aphis nerii* | *Nerium oleander* | - |
| 10Md 72 | Portugal | Madeira | Aphidinae | Aphidini | *Aphis nerii* | NI | - |
| 09Md 66 | Portugal | Madeira | Aphidinae | Aphidini | *Aphis pomi* | *Eriobotrya japonica* | - |
| 10Md 195 | Portugal | Madeira | Aphidinae | Aphidini | *Aphis pomi* | *Eriobotrya japonica* | - |
| 10Md 196 | Portugal | Madeira | Aphidinae | Aphidini | *Aphis ruborum* | *Rubus* sp. | - |
| 09Md 13 | Portugal | Madeira | Aphidinae | Aphidini | *Aphis solanella* | *Solanum nigrum* | - |
| 10Md 160 | Portugal | Madeira | Aphidinae | Aphidini | *Aphis solanella* | *Solanum nigrum* | - |
| 10Md 99 | Portugal | Madeira | Aphidinae | Aphidini | *Aphis spiraecola* | *Bidens pilosa* | - |
| 09Md 55 | Portugal | Madeira | Aphidinae | Aphidini | *Aphis spiraecola* | *Cotoneaster* sp. | - |
| 09Md 65 | Portugal | Madeira | Aphidinae | Aphidini | *Aphis spiraecola* | NI | - |
| 10Md 131 | Portugal | Madeira | Aphidinae | Aphidini | *Aphis spiraecola* | *Asteraceae* | - |
| 10Md 163 | Portugal | Madeira | Aphidinae | Aphidini | *Aphis spiraecola* | *Erica* sp. | - |
| 09Md 8 | Portugal | Madeira | Aphidinae | Aphidini | *Aphis ulicis* | *Ulex europaeus* | - |
| 10Md 127 | Portugal | Madeira | Aphidinae | Aphidini | *Aphis ulicis* | *Ulex europaeus* | - |
| 09Md 44 | Portugal | Madeira | Aphidinae | Aphidini | *Aphis umbrella* | *Lavatera critica* | - |
| 10Md 83 | Portugal | Madeira | Aphidinae | Aphidini | *Aphis* *umbrella* | *Malva* sp | - |
| 09Md 58 | Portugal | Madeira | Aphidinae | Aphidini | *Aphis* sp. | *Agapanthus* sp. | - |
| 10Md 110 | Portugal | Madeira | Aphidinae | Aphidini | *Aphis sp.* | *Centaurium* sp. | - |
| 10Md 120 | Portugal | Madeira | Aphidinae | Aphidini | *Aphis* sp. | *Centaurium* sp. | - |
| 09Md 27 | Portugal | Madeira | Aphidinae | Aphidini | *Aphis* sp. | *Nerium oleander* | - |
| 09Md 43 | Portugal | Madeira | Aphidinae | Aphidini | *Aphis* sp. | *Nerium oleander* | - |
| 10Md 140 | Portugal | Madeira | Aphidinae | Aphidini | *Aphis* sp. | *Rubia fruticosa* | - |
| 09Md 11 | Portugal | Madeira | Aphidinae | Aphidini | *Aphis* sp. | *Rubus* sp. | - |
| 09Md 40 | Portugal | Madeira | Aphidinae | Aphidini | *Aphis* sp. | NI | - |
| 10Md 91 | Portugal | Madeira | Aphidinae | Macrosiphini | *Aulacorthum solani* | Labiatae | - |
| 11Md 199 | Portugal | Madeira | Aphidinae | Macrosiphini | *Aulacorthum solani* | *Euphorbia piscatoria* | + |
| 10Md 144 | Portugal | Madeira | Aphidinae | Macrosiphini | *Brachycaudus cardui* | NI | - |
| 11Md 201 | Portugal | Madeira | Aphidinae | Macrosiphini | *Brachycaudus cardui* | *Galactites tomentosa* | - |
| 10Md 80 | Portugal | Madeira | Aphidinae | Macrosiphini | *Brachycaudus helichrysi* | *Euryops pectinatus* | - |
| 10Md 81 | Portugal | Madeira | Aphidinae | Macrosiphini | *Brachycaudus helichrysi* | *Erodium* sp | - |
| 10Md 193 | Portugal | Madeira | Aphidinae | Macrosiphini | *Brachycaudus schwartzi* | *Prunus domestica* | - |
| 10Md 137 | Portugal | Madeira | Aphidinae | Macrosiphini | *Cavariella aegopodii* | *Vicia sativa* | - |
| 10Md 138 | Portugal | Madeira | Aphidinae | Macrosiphini | *Cavariella aegopodii* | *Ammi majus* | - |
| 10Md 148 | Portugal | Madeira | Aphidinae | Macrosiphini | *Cavariella theobaldi* | NI | - |
| 09Md 60 | Portugal | Madeira | Aphidinae | Macrosiphini | *Eucarazzia elegans* | *Mentha* sp | - |
| 09Md 50 | Portugal | Madeira | Aphidinae | Macrosiphini | *Hyperomyzus lactucae* | *Asteraceae* | - |
| 10Md 139 | Portugal | Madeira | Aphidinae | Macrosiphini | *Hyperomyzus lactucae* | *Asteraceae* | - |
| 10Md 103 | Portugal | Madeira | Aphidinae | Macrosiphini | *Hyperomyzus lactucae* | *Crepis divaricata* | - |
| 10Md 130 | Portugal | Madeira | Aphidinae | Macrosiphini | *Hyperomyzus lactucae* | *Crepis divaricata* | - |
| 10Md 93 | Portugal | Madeira | Aphidinae | Macrosiphini | *Hyperomyzus lactucae* | *Sonchus spp.* | - |
| 09Md 9 | Portugal | Madeira | Aphidinae | Macrosiphini | *Macrosiphoniella madeirensis* | *Elicrisum* | - |
| 11Md 203 | Portugal | Madeira | Aphidinae | Macrosiphini | *Macrosiphum euphorbiae* | *Solandra grandiflora* | + |
| 10Md 147 | Portugal | Madeira | Aphidinae | Macrosiphini | *Macrosiphum euphorbiae* | *Solanum tuberosum* | - |
| 10Md 176 | Portugal | Madeira | Aphidinae | Macrosiphini | *Macrosiphum rosae* | *Rosa* sp.*.* | - |
| 10Md 108 | Portugal | Madeira | Aphidinae | Macrosiphini | *Metopolophium festucae* | *Lagurus ovatus* | - |
| 10Md 97 | Portugal | Madeira | Aphidinae | Macrosiphini | *Metopolophium dirhodum* | *Rosa* sp. | - |
| 10Md 190 | Portugal | Madeira | Aphidinae | Macrosiphini | *Nasonovia ribisnigri* | NI | - |
| 10Md 76 | Portugal | Madeira | Aphidinae | Macrosiphini | *Neomyzus circumflexus* | *Zantedeschia aethiopicaca* | - |
| 10Md 95 | Portugal | Madeira | Aphidinae | Macrosiphini | *Neotoxoptera formosana* | *Allium triquetrum* | - |
| 10Md 82 | Portugal | Madeira | Aphidinae | Aphidini | *Rhopalosiphum maidis* | *Poacea* | - |
| 09Md 112 | Portugal | Madeira | Aphidinae | Aphidini | *Rhopalosiphum padi* | *Avena barbata* | - |
| 10Md 156 | Portugal | Madeira | Aphidinae | Aphidini | *Rhopalosiphum padi* | *Poacea* | - |
| 10Md 77 | Portugal | Madeira | Aphidinae | Macrosiphini | *Toxoptera aurantii* | *Citrus limon* | - |
| 10Md 187 | Portugal | Madeira | Aphidinae | Macrosiphini | *Toxoptera citricidus* | *Annonaceae* | + |
| 10Md 117 | Portugal | Madeira | Aphidinae | Macrosiphini | *Uroleucon hypochoeridis* | *Crepis divaricata* | - |
| 10Md 124 | Portugal | Madeira | Aphidinae | Macrosiphini | *Uroleucon jaceae* | *Carthamus lanatus* | - |
| 10Md 125 | Portugal | Madeira | Aphidinae | Macrosiphini | *Uroleucon picridis* | *Helminthotheca echioides* | - |
| 10Md 86 | Portugal | Madeira | Aphidinae | Macrosiphini | *Uroleucon sonchi* | *Sonchus* sp. | - |
| 10Md 102 | Portugal | Madeira | Aphidinae | Macrosiphini | *Uroleucon sonchi* | *Crepis divaricata* | - |
| 10Md 78 | Portugal | Madeira | Aphidinae | Macrosiphini | *Uroleucon* sp. | NI | - |
| 10Md 197 | Portugal | Madeira | Aphidinae | Macrosiphini | *Uroleucon* sp. | *Asteraceae* | - |
| 10Md 73 | Portugal | Madeira | Aphidinae | Macrosiphini | *Wahlgreniella arbuti* | NI | - |
| 10Md 166 | Portugal | Madeira | Aphidinae | Aphidini | *Aphis solanella* | *Solanum nigrum* | - |
| BS_Val 23 | Spain | Valencia | Aphidinae | Aphidini | *Aphis fabae* | *Scolymus* sp. | - |
| BS_Val 6 | Spain | Valencia | Aphidinae | Aphidini | *Aphis nerii* | *Nerium oleander* | + |
| BS_Val 22 | Spain | Valencia | Aphidinae | Aphidini | *Aphis* sp. | *Gossypium* sp. | - |
| BS_Val 8 | Spain | Valencia | Aphidinae | Aphidini | *Aphis* sp. | *Genista* sp. | + |
| BS_Val 20 | Spain | Valencia | Aphidinae | Aphidini | *Aphis urticata* | *Fleurya aestuans* | - |
| CS_Val | Spain | Valencia | Aphidinae | Macrosiphini | *Cavariella* sp. | *Salix* sp. | + |
| BS_Val 1 | Spain | Valencia | Aphidinae | Macrosiphini | *Cavariella* sp. | *Salix* sp. | + |
| BS_Val 21 | Spain | Valencia | Aphidinae | Macrosiphini | *Liosomaphis berberidis* | *Berberis vulgaris* | - |
| BS_Val 15 | Spain | Valencia | Aphidinae | Macrosiphini | *Macrosiphoniella absinthii* | *Artemisia absinthium* | - |
| GRA34 | Greece | Etoloakarnania | Chaitophorinae | Chaitophorini | *Chaitophorus populialbae* | *Populus* sp. | - |
| GRC204 | Greece | Etoloakarnania | Chaitophorinae | Chaitophorini | *Chaitophorus populialbae* | *Populus alba* | - |
| GRB142 | Greece | Etoloakarnania | Chaitophorinae | Chaitophorini | *Chaitophorus leucomelas* | *Populus nigra* | - |
| GRC201 | Greece | Etoloakarnania | Chaitophorinae | Chaitophorini | *Sipha maydis* | *Zea mays* | + |
| 10Az4 | Portugal | Terceira (Azores) | Pterocommatinae | *-* | *Pterocomma* sp. | NI | - |
| GRB148 | Greece | Etoloakarnania | Myzocallidinae | Calaphidini | *Calaphis juglandis* | *Juglans regia* | - |
| GRA51 | Greece | Etoloakarnania | Myzocallidinae | Myzocallidini | *Chromaphis juglandicola* | *Juglans regia* | - |
| GRB146 | Greece | Etoloakarnania | Myzocallidinae | Myzocallidini | *Chromaphis juglandicola* | *Juglans regia* | - |
| GRA36 | Greece | Etoloakarnania | Myzocallidinae | Myzocallidini | *Eucallipterus tiliae* | *Tilia* sp. | - |
| GRC236 | Greece | Etoloakarnania | Myzocallidinae | Myzocallidini | *Eucallipterus tiliae* | *Tillia* sp. | - |
| GRA79 | Greece | Etoloakarnania | Myzocallidinae | Myzocallidini | *Hoplocallis picta* | *Arum* sp. | - |
| GRC222 | Greece | Etoloakarnania | Myzocallidinae | Myzocallidini | *Hoplocallis picta* | *Quercus ilex* | - |
| GrB149 | Greece | Etoloakarnania | Myzocallidinae | Myzocallidini | *Myzocallis custanicola* | *Castanea sativa* | - |
| GRC206 | Greece | Etoloakarnania | Myzocallidinae | Myzocallidini | *Monellia caryella* | *Carya illinoensis* | - |
| GRC212 | Greece | Etoloakarnania | Myzocallidinae | Myzocallidini | *Myzocallis coryli* | *Corylus avellanae* | - |
| GRA46 | Greece | Etoloakarnania | Myzocallidinae | Myzocallidini | *Panaphis juglandis* | *Juglans regia* | - |
| GRB113 | Greece | Etoloakarnania | Myzocallidinae | Siphini | *Sipha* sp. | *Alopecurus myosuroides* | - |
| GRC230 | Greece | Etoloakarnania | Myzocallidinae | Myzocallidini | *Therioaphis trifolli* | *Medicago sativa* | - |
| GRC237 | Greece | Etoloakarnania | Myzocallidinae | Myzocallidini | *Tinocallis sp.* | *Ulmus americana* | - |
| 10Md 170 | Portugal | Madeira | Chaitophorinae | Siphini | *Sipha* sp. | *Poacea* | - |
| 10Md 171 | Portugal | Madeira | Chaitophorinae | Siphini | *Sipha* sp. | *Avena* sp. | - |
| 10Md 194 | Portugal | Madeira | Myzocallidinae | Myzocallidini | *Tuberculoides annulatus* | *Quercus* sp. | - |
| 10Az1 | Portugal | Terceira (Azores) | Drepanosiphinae | *-* | *Drepanosiphum* sp. | NI | - |
| 10AzG3 | Portugal | São Miguel (Azores) | Drepanosiphinae | *-* | *Neophyllaphis podocarpi* | *Podocarpus macrophylus* | + |
| 10Md 74 | Portugal | Madeira | Myzocallidinae | Myzocallidini | *Myzocallis kuricola* | NI | - |
| 09Md 62 | Portugal | Madeira | Myzocallidinae | Myzocallidini | *Takecallis* sp. | NI | - |
| BS_Val 14 | Spain | Valencia | Myzocallidinae | Myzocallidini | *Hoplocallis picta* | *Quercus ilex* | - |
| BS_Val 13 | Spain | Valencia | Myzocallidinae | Myzocallidini | *Panaphis juglansdis* | *Juglans regia* | - |
| BS_Val 10 | Spain | Valencia | Chaitophorinae | Chaitophorini | *Chaitophorus* sp. | *Populus nigra* | - |
| BS_Val 11 | Spain | Valencia | Thelaxinae | *-* | *Thelaxes suberi* | *Quercus ilex* | - |
| GRB111 | Greece | Etoloakarnania | Lachninae | Eulachnini | *Cinara* sp. | *Plaatycladus* sp. | - |
| GRA68 | Greece | Etoloakarnania | Lachninae | Lachnini | *Pterochloroides persicae* | *Prunus persica* | - |
| 09Ir3 | Iran | Tehran | Lachninae | Eulachnini | *Cinara* sp. | *Pinus* sp. | + |
| 10Ir12 | Iran | Tehran | Lachninae | Eulachnini | *Cinara* sp. | *Cupressus* sp.*.* | + |
| BS_Is(CCeI -) | Israel | - | Lachninae | Eulachnini | *Cinara cedri* | *Cedrus* sp. | + |
| BS_Is(CceI^+^) | Israel | - | Lachninae | Eulachnini | *Cinara cedri* | *Cedrus* sp. | + |
| 09Md 23 | Portugal | Madeira | Lachninae | Eulachnini | *Cinara fresai* | *Cupressus macrocarpa* | + |
| 10Md 180 | Portugal | Madeira | Lachninae | Eulachnini | *Cinara juniperi* | *Juniperus cedrus* | - |
| 09Md 16 | Portugal | Madeira | Lachninae | Eulachnini | *Cinara maritimae* | *Pinus* sp. | - |
| 09Md 18 | Portugal | Madeira | Lachninae | Eulachnini | *Cinara maritimae* | *Pinus pinaster* | - |
| 09Md 48 | Portugal | Madeira | Lachninae | Eulachnini | *Cinara pinea* | *Pinus* sp. | + |
| 10Md 181 | Portugal | Madeira | Lachninae | Eulachnini | *Cinara pinea* | *Pinus* sp. | + |
| 10Md 167 | Portugal | Madeira | Lachninae | Eulachnini | *Cinara pinihabitans* | *Pinus* sp. | - |
| 09Md 26 | Portugal | Madeira | Lachninae | Eulachnini | *Cinara tujafilina* | *Cupressus* sp. | - |
| 09Md 70 | Portugal | Madeira | Lachninae | Eulachnini | *Cinara* sp. | *Cupressus* sp. | - |
| 10Md 79 | Portugal | Madeira | Lachninae | Eulachnini | *Cinara* sp. | *Cupressus* sp. | - |
| 10Md 162 | Portugal | Madeira | Lachninae | Eulachnini | *Cinara* sp. | *Pinus sp.* | - |
| 09Md 49 | Portugal | Madeira | Lachninae | Eulachnini | *Eulachnus mediterraneus* | *Pinus* sp. | - |
| 09Md 15 | Portugal | Madeira | Lachninae | Eulachnini | *Eulachnus rileyi* | *Picea* sp. | - |
| 09Md 24 | Portugal | Madeira | Lachninae | Lachnini | *Tuberolachnus salignus* | *Salix canariensis* | + |
| BS_Ter (Lro) | Spain | Teruel | Lachninae | Lachnini | *Lachnus roboris* | *Quercus* sp. | - |
| BS_Gal(CCeG) | Spain | Galicia | Lachninae | Eulachnini | *Cinara cedri* | *Cedrus* sp. | + |
| BS_Sal(CCeS) | Spain | Salamanca | Lachninae | Eulachnini | *Cinara cedri* | *Cedrus* sp. | + |
| BS_Tar (CCeT) | Spain | Tarancona | Lachninae | Eulachnini | *Cinara cedri* | *Cedrus* sp. | + |
| BS_Ter (Cpia) | Spain | Teruel | Lachninae | Eulachnini | *Cinara sp.* | *Cedrus* sp. | - |
| BS_Ter (Cju) | Spain | Teruel | Lachninae | Eulachnini | *Cinara juniperi* | *Juniperus oxycedrus* | - |
| BS_Ter (CpI) | Spain | Teruel | Lachninae | Eulachnini | *Cinara pilicornis* | *Picea* sp. | - |
| BS_Ter (Csc) | Spain | Teruel | Lachninae | Eulachnini | *Cinara schimitscheki* | *Pinus nigra* | - |
| BS_Ter (Cgu) | Spain | Teruel | Lachninae | Eulachnini | *Cinara gudaris* | *Pinus nigra* | - |
| BS_Ter (Cpn) | Spain | Teruel | Lachninae | Eulachnini | *Cinara pinimaritimae* | *Pinus pinaster* | - |
| BS_Vald(CCeVld) | Spain | Valdelinares | Lachninae | Eulachnini | *Cinara cedri* | *Cedrus* sp. | - |
| AS_Val (CCeV) | Spain | Valencia | Lachninae | Eulachnini | *Cinara cedri* | *Cedrus* sp. | + |
| CS_CceV | Spain | Valencia | Lachninae | Eulachnini | *Cinara cedri* | *Cedrus* sp. | + |
| BS_Zar (CCeZ) | Spain | Zaragoza | Lachninae | Eulachnini | *Cinara cedri* | *Cedrus* sp. | + |
| CS_Val 12 | Spain | Valencia | Lachninae | Eulachnini | *Cinara cupressi* | *Cupressus sempervirens* | - |
| CS_Val 3 | Spain | Valencia | Lachninae | Eulachnini | *Cinara juniperi* | *Juniperus communis* | + |
| BS_Val (Cma) | Spain | Valencia | Lachninae | Eulachnini | *Cinara maghrebica* | *Pinus halepensis* | - |
| CS_Val 2 | Spain | Valencia | Lachninae | Eulachnini | *Cinara maritimae* | *Pinus pinaster* | + |
| CS_Val 5 | Spain | Valencia | Lachninae | Eulachnini | *Cinara pinea* | *Pinus sylvestris* | - |
| CS_Val 4 | Spain | Valencia | Lachninae | Eulachnini | *Cinara pinea* | *Pinus sylvestris* | + |
| BS_Val (Ctuj) | Spain | Valencia | Lachninae | Eulachnini | *Cinara tujafilina* | *Platycladus orientalis* | - |
| CS_Val 7 | Spain | Valencia | Lachninae | Eulachnini | *Cinara tujafilina* | *Platycladus orientalis* | + |
| BS_Val (Msu) | Spain | Valencia | Lachninae | Lachnini | *Maculolachnus submaculata* | *Rosa* sp. | + |
| BS_Val (Tsa) | Spain | Valencia | Lachninae | Lachnini | *Tuberolachnus salignus* | *Salix* sp. | + |
| CS_Val 9 | Spain | Valencia | Lachninae | Lachnini | *Tuberolachnus salignus* | *Salix* sp. | + |
| GRC200 | Greece | Etoloakarnania | Mindarinae | *-* | *Mindarus abietinus* | *Abies* sp. | - |
| GRA47 | Greece | Etoloakarnania | Eriosomatinae | Eriosomatini | *Eriosoma lanuginosum* | *Ulmus* sp. | - |
| GRC282 | Greece | Etoloakarnania | Eriosomatinae | Eriosomatini | *Eriosoma lanuginosum* | *Ulmus* sp. | - |
| GRA54 | Greece | Etoloakarnania | Eriosomatinae | Eriosomatini | *Kaltenbachiella pallida* | *Ulmus* sp. | - |
| GRC221 | Greece | Etoloakarnania | Eriosomatinae | Eriosomatini | *Tetraneura akinirae* | *Ulmus* sp. | - |
| GRA69 | Greece | Etoloakarnania | Eriosomatinae | Fordini | *Baizongia pistaciae* | *Pistacia terebinthus* | + |
| GRA64 | Greece | Etoloakarnania | Eriosomatinae | Fordini | *Forda formicaria* | *Pistacia terebinthus* | - |
| GRC211 | Greece | Etoloakarnania | Eriosomatinae | Fordini | *Forda formicaria* | *Ulmus* sp. | - |
| GRA67 | Greece | Etoloakarnania | Eriosomatinae | Fordini | *Geoica utricularia* | *Pistacia terebinthus* | - |
| CS_Val 16 | Spain | Valencia | Eriosomatinae | Fordini | *Geoica utricularia* | *Pistacia terebinthus* | - |
| CS_Val 17 | Spain | Valencia | Eriosomatinae | Fordini | *Forda formicaria* | *Pistacia terebinthus* | - |
| CS_Val 18 | Spain | Valencia | Eriosomatinae | Fordini | *Forda marginata* | *Pistacia terebinthus* | - |
| GRA53 | Greece | Etoloakarnania | Erisoomatinae | Pemphigini | *Pemphigus bursarius* | *Populus nigra* | - |
| GRB154 | Greece | Etoloakarnania | Eriosomatinae | Pemphigini | *Pemphigus bursarius* | *Populus nigra* | - |
| GRC225 | Greece | Etoloakarnania | Eriosomatinae | Pemphigini | *Pemphigus bursarius* | *Populus nigra* | - |
| GRB153 | Greece | Etoloakarnania | Eriosomatinae | Pemphigini | *Pemphigus spyrothecae* | *Populus nigra* | - |
| GRA24 | Greece | Etoloakarnania | Eriosomatinae | Pemphigini | *Prociphilus oleae* | *Olea europea* | - |
| GRC217 | Greece | Etoloakarnania | Eriosomatinae | Pemphigini | *Prociphilus oleae* | *Olea europea* | - |
| CS_Val 19 | Spain | Valencia | Eriosomatinae | Pemphigini | *Pemphigus spyrothecae* | *Populus nigra* | - |
| BS_Ter | Spain | Teruel | Eriosomatinae | Pemphigini | *Pemphigus spyrothecae* | *Populus nigra* | - |

*: The nomenclature is according to (Remaudiere, G. and M. Remaudiere, 1997). Catalogue des Aphididae du monde. Institut National de la Recherche Agronomique, Paris, France.

GRA: collected in 2006

GRB: collected in 2008

GRC: collected in 2009

09Md/Ir: collected in 2009

10Md/Ir/Az: collected in 2010

11Md: collected in 2011

AS_:collected in 2003

BS_: collected in 2005

CS_: collected in 2009

NI: not identified
