# Supplementary material for: Detection and Characterization of Wolbachia Infections in Natural Populations of Aphids: Is the Hidden Diversity Fully Unraveled?
Source: PLoS One. 2011 Dec 13;6(12):e28695. doi: 10.1371/journal.pone.0028695 (PMC3236762; doi:10.1371/journal.pone.0028695)
Supplement: Table S2 — Taxonomic details of Wolbachia hosts and accession numbers of analyzed sequences. (DOC) [file pone.0028695.s007.doc]

Table S2. Taxonomic details of *Wolbachia* hosts and accession numbers of analyzed sequences.

|  |  |  |  | GenBank accession number | | | |  |
| --- | --- | --- | --- | --- | --- | --- | --- | --- |
| **Host species** | **Phylum** | **Class** | **Order** | **16S rRNA** | ***fts*Z** | ***groEL*** | ***gltA*** | **Supergroup** |
| *Drosophila melanogaster* | Arthropoda | Insecta | Diptera | AE017196 | AE017196 | AE017196 | AE017196 | A |
| *Drosophila simulans* | Arthropoda | Insecta | Diptera | CP001391 | CP001391 | CP001391 | CP001391 |
| *Nasonia giraulti* | Arthropoda | Insecta | Hymenoptera | M84690 | U28182 | AY714810 | AY714793 |
| *Nasonia vitripennis* | Arthropoda | Insecta | Hymenoptera | M84688 | U28188 | AY714812 | AY714795 |
| *Nasonia longicornis* | Arthropoda | Insecta | Hymenoptera | M84691 |  | AY714811 | AY714794 |  |
| *Nasonia vitripennis* | Arthropoda | Insecta | Hymenoptera | M84686 | U28205 | AY714796 | AY714782 | B |
| *Encarsia formosa* | Arthropoda | Insecta | Hymenoptera | AF045189 | U28196 | AY714797 | AY714783 |
| *Bryobia* sp*.* I | Arthropoda | Prostigmata | Acarina | EU499318 | EU499323 | EU499333 | EU499328 |  |
| *Bryobia praetiosa* | Arthropoda | Prostigmata | Acarina | EU499317 | EU499322 | EU499332 | EU499327 |  |
| *Tetranychus urticae* | Arthropoda | Prostigmata | Acarina | EU499319 | EU499324 | EU499334 | EU499329 |  |
| *Bryobia sarothamni* | Arthropoda | Prostigmata | Acarina | EU499315 | EU499320 | EU499330 | EU499325 |  |
| *Dirofilaria immitis* | Nematoda | Secernentea | Spirurida | Z49261 | AJ010272 | AJ558023 | AJ609641 | C |
| *Dirofilaria repens* | Nematoda | Secernentea | Spirurida | AJ276500 | AJ010273 | AJ609653 | - |
| *Onchocerca gibsoni* | Nematoda | Secernentea | Spirurida | AJ276499 | AJ270267 | AJ609652 | AJ609639 |
| *Onchocerca ochengi* | Nematoda | Secernentea | Spirurida | AJ010276 | AJ010266 | - | AJ609640 |  |
| *Brugia malayi* | Nematoda | Secernentea | Spirurida | AE017321 | AE017321 | AE017321 | AJ609643 | D |
| *Brugia pahangi* | Nematoda | Secernentea | Spirurida | AJ012646 | AJ010270 | AJ609654 | AJ609642 |
| *Wuchereria bancrofti* | Nematoda | Secernentea | Spirurida | AF093510 | AF081198 | - | AJ609644 |  |
| *Litomosoides brasiliensis* | Nematoda | Secernentea | Spirurida | AJ548799 | - | AJ609655 | AJ609646 |  |
| *Litomosoides sigmodontis* | Nematoda | Secernentea | Spirurida | AF069068 | AJ010271 | AF409113 | AJ609645 |  |
| *Folsomia candida* | Arthropoda | Collembola | Collembola | AF179630 | AJ344216 | — | AJ609649 | E |
| *Mesaphorura italica* | Arthropoda | Collembola | Collembola | AJ575104 | AJ575103 | — | — |
| *Kalotermes flavicollis* | Arthropoda | Insecta | Isoptera | Y11377 | AJ292345 | AJ609660 | AJ609651 | F |
| *Mansonella ozzardi.* | Nematoda | Secernentea | Spirurida | AJ279034 | - | AJ609657 | AJ609647 |
| *Rhinocyllus conicus* | Arthropoda | Insecta | Coleoptera | M85267 | - |  |  |  |
| *Microcerotermes*sp. | Arthropoda | Insecta | Isoptera | AJ292347 | AJ292346 | AJ628411 |  |  |
| *Myrmeleon mobilis* | Arthropoda | Insecta | Neuroptera | DQ068882 | - | - |  |  |
| *Zootermopsis angusticollis* | Arthropoda | Insecta | Isoptera | AY764279 | AY764283 | AY764278 | AY764281 | H |
| *Zootermopsis nevadensis* | Arthropoda | Insecta | Isoptera | AY764280 | AY764284 | AY764277 | AY764282 |
| *Ctenocephalides felis* | Arthropoda | Insecta | Siphonaptera | AY335923 | AJ628415 | AJ609659 | AJ609650 | I |
| *Orchopeas leucopus* | Arthropoda | Insecta | Siphonaptera | AY335924 |  |  |  |  |
| *Dipetalonema gracile* | Nematoda | Secernentea | Spirurida | AJ548802 |  | AJ609658 | AJ609648 | J |
| *Bryobia* sp. | Arthropoda | Arachnida | Prostigmata | EU499316 | EU499321 | EU499331 | EU499326 | K |
| *Radopholus similis* | Nematoda | Chromadorea | Rhabditida | EU833482 | EU833483 | EU833484 | - | L |
|  |  |  |  |  |  |  |  |  |
