# Supplementary material for: Detection and Characterization of Wolbachia Infections in Natural Populations of Aphids: Is the Hidden Diversity Fully Unraveled?
Source: PLoS One. 2011 Dec 13;6(12):e28695. doi: 10.1371/journal.pone.0028695 (PMC3236762; doi:10.1371/journal.pone.0028695)
Supplement: Table S3 — Estimates of Evolutionary Divergence (average) over Sequence Pairs between and within Wolbachia Supergroups. (DOC) [file pone.0028695.s008.doc]

Table S3. Estimates of Evolutionary Divergence (average) over Sequence Pairs between and within *Wolbachia* Supergroups

|  | A | B | C | D | E | F | H | I | J | K | L | **Supergroup_M** | **Supergroup_N** | Supergroup_A Aphids | Supergroup_B Aphids |
| --- | --- | --- | --- | --- | --- | --- | --- | --- | --- | --- | --- | --- | --- | --- | --- |
| Supergroup_A | 0.003 |  |  |  |  |  |  |  |  |  |  |  |  |  |  |
| Supergroup_B | 0.036 | 0.006 |  |  |  |  |  |  |  |  |  |  |  |  |  |
| Supergroup_C | 0.034 | 0.062 | 0.018 |  |  |  |  |  |  |  |  |  |  |  |  |
| Supergroup_D | 0.021 | 0.048 | 0.037 | 0.01 |  |  |  |  |  |  |  |  |  |  |  |
| Supergroup_E | **0.014** | 0.047 | 0.041 | 0.029 | 0.01 |  |  |  |  |  |  |  |  |  |  |
| Supergroup_F | **0.017** | 0.042 | 0.034 | 0.021 | 0.025 | 0.008 |  |  |  |  |  |  |  |  |  |
| Supergroup_H | **0.017** | 0.047 | 0.044 | 0.029 | **0.017** | 0.027 | 0 |  |  |  |  |  |  |  |  |
| Supergroup_I | 0.036 | 0.068 | 0.065 | 0.052 | 0.046 | 0.048 | 0.051 | 0.003 |  |  |  |  |  |  |  |
| Supergroup_J | 0.042 | 0.062 | 0.031 | 0.042 | 0.049 | 0.039 | 0.052 | 0.072 | n/c |  |  |  |  |  |  |
| Supergroup_K | 0.031 | 0.051 | 0.044 | 0.037 | 0.035 | 0.032 | 0.033 | 0.062 | 0.049 | n/c |  |  |  |  |  |
| Supergroup_L | 0.043 | 0.068 | 0.058 | 0.048 | 0.051 | 0.051 | 0.049 | 0.076 | 0.06 | 0.044 | n/c |  |  |  |  |
| **Supergroup_M** | 0.033 | 0.021 | 0.058 | 0.044 | 0.039 | 0.04 | 0.041 | 0.065 | 0.059 | 0.041 | 0.058 | 0.013 |  |  |  |
| **Supergroup_N** | 0.024 | 0.042 | 0.04 | 0.03 | 0.031 | 0.026 | 0.03 | 0.054 | 0.043 | 0.022 | 0.031 | 0.03 | 0.002 |  |  |
| Supergroup_A Aphids | 0.002 | 0.036 | 0.033 | 0.02 | 0.012 | 0.017 | 0.016 | 0.034 | 0.041 | 0.029 | 0.041 | 0.032 | 0.022 | 0 |  |
| Supergroup_B Aphids | 0.034 | 0.009 | 0.062 | 0.046 | 0.044 | 0.043 | 0.045 | 0.067 | 0.063 | 0.049 | 0.066 | 0.011 | 0.04 | 0.034 | 0 |

n/c: not calculated (Supergroups J, K and L are represented by a single sequence)
